# Similarity of Cortical Activity Patterns Predicts *generalization* Behavior

**DOI:** 10.1371/journal.pone.0078607

**Published:** 2013-10-16

**Authors:** Crystal T. Engineer, Claudia A. Perez, Ryan S. Carraway, Kevin Q. Chang, Jarod L. Roland, Andrew M. Sloan, Michael P. Kilgard

**Affiliations:** School of Behavioral and Brain Sciences, The University of Texas at Dallas, Richardson, Texas, United States of America; University of Salamanca- Institute for Neuroscience of Castille and Leon and Medical School, Spain

## Abstract

Humans and animals readily generalize previously learned knowledge to new situations. Determining similarity is critical for assigning category membership to a novel stimulus. We tested the hypothesis that category membership is initially encoded by the similarity of the activity pattern evoked by a novel stimulus to the patterns from known categories. We provide behavioral and neurophysiological evidence that activity patterns in primary auditory cortex contain sufficient information to explain behavioral categorization of novel speech sounds by rats. Our results suggest that category membership might be encoded by the similarity of the activity pattern evoked by a novel speech sound to the patterns evoked by known sounds. Categorization based on featureless pattern matching may represent a general neural mechanism for ensuring accurate generalization across sensory and cognitive systems.

## Introduction

When faced with a sensory stimulus that could indicate a predator, prey, or a mate, accurate generalization is critical for survival [1]. For example, vervet monkeys learn to emit different warning calls for each class of predator in their environment, and monkeys who hear these calls exhibit distinct behaviors that indicate they understand the category that each type of call represents [2]. Humans and animals possess the remarkable ability to quickly and accurately determine how similar any image, sound, or smell is to previously learned stimuli. 

The first step in categorizing a novel stimulus appears to be quantifying its similarity to known category members [3,4]. Many studies have documented the presence of a generalization gradient for stimuli varying along a single dimension. Pigeons trained to peck a colored light also respond to colors of similar wavelength [5]. Following conditioning to a tone, both humans and animals respond to tones of similar frequency and respond less as similarity decreases [6,7]. However, physical similarity does not always predict perceptual similarity even for stimuli that vary along a single dimension [8-10]. 

The similarity of real world stimuli is notoriously difficult to predict. The consonants /d/ and /t/ (as in “dad and “tad”) have different voice onset times, pitch contours, formant transition durations, formant onset frequencies, F1 cutbacks, and burst intensities [11]. Male and female voices have different pitches, levels of breathiness, formant frequencies, and formant amplitudes [12]. Any of these features is sufficient to distinguish between ambiguous sounds, but none of these features is necessary to identify the phoneme or gender [13-15]. The so-called “lack of invariance” problem in speech perception also occurs in face perception [16,17]. Dozens of physical differences, including pupil to pupil distance, chin shape, and nose length, can be used to distinguish between faces but no single feature or set of features is required. Modern face recognition algorithms use template matching because feature-based approaches failed to support robust recognition [18].

Commercial systems for speech and music recognition have also abandoned the use of feature-based approaches [19,20], but psychophysical and neurophysiological studies continue to focus on the representation of a small set of speech features [21-24]. In this study, we test the hypothesis that the similarity of activity patterns in sensory cortex supports effective speech sound categorization without the need to compute a set of particular acoustic features. Our study provides a direct demonstration that, like face recognition, featureless template matching accounts for speech categorization performance. 

## Materials and Methods

Twenty-one rats were trained to categorize speech sounds by voicing or gender. We trained rats to press a lever in response to a single speech sound and refrain from lever pressing to a second speech sound. We then tested their ability to generalize to novel speech sounds. Half of the rats in our study were trained to categorize sounds based on speaker gender (female vs. male, Gender Task group), while the other half were trained to categorize speech sounds based on voicing (‘dad’ vs. ‘tad’, Voicing Task group). Behavioral performance on four generalization tasks was compared to multiunit activity recorded at 441 primary auditory cortex (A1) sites from eleven experimentally naïve rats and 903 A1 sites from twenty-one speech trained rats (female Sprague Dawley rats were obtained from Charles River Laboratories). Our datasets are freely available upon request. Behavioral training and A1 recording procedures are identical to our previous studies [25,26]. 

### Ethics statement

This study was performed in strict accordance with the recommendations in the Guide for the Care and Use of Laboratory Animals of the National Institutes of Health. The protocol was approved by The University of Texas at Dallas Institutional Animal Care and Use Committee (Protocol Number: 99-06). All surgery was performed under sodium pentobarbital anesthesia, and every effort was made to minimize suffering.

### Speech stimuli

**Figure 1:**
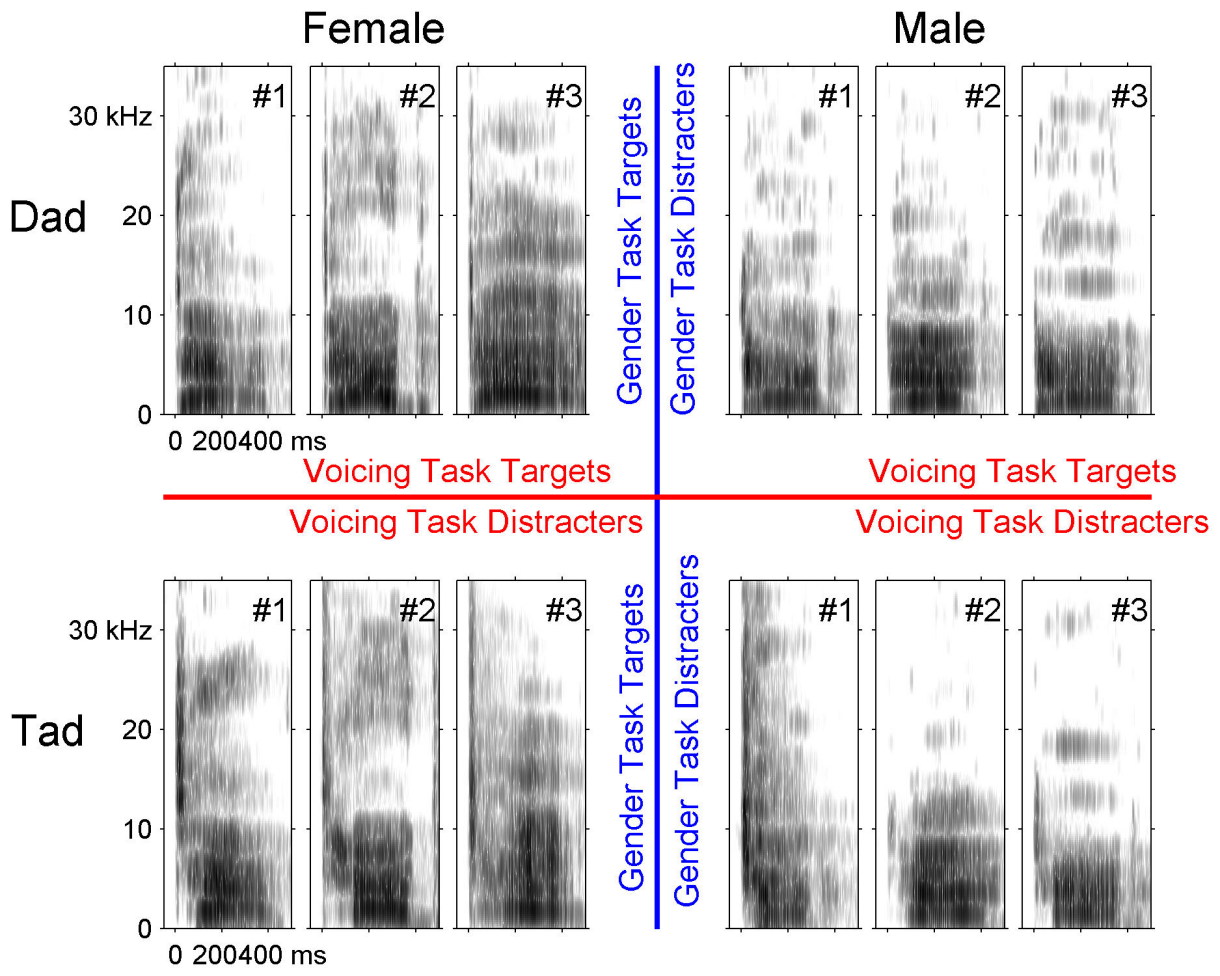
Spectrograms of each speech sound grouped by gender and voicing. Rows differ in voicing (top row is ‘dad’, bottom row is ‘tad’), while columns differ in gender (left three columns are female, right three columns are male). Frequency is represented on the *y* axis (0–35 kHz) and time on the *x* axis (-50 to 500 ms). Speech sounds were shifted one octave higher to accommodate the rat hearing range.

The stimulus set for these experiments was designed so that each sound can be categorized based on (1) the gender of the speaker or (2) the voicing of the initial dental consonants (/d/ vs. /t/). We used the voiced word ‘dad’ and the voiceless word ‘tad’ spoken in isolation by 3 male and 3 female native English speakers (*n* = 12 sounds, Figure 1). The sound names were shortened in the figures; for example, ‘DM3’ refers to the sound ‘dad’ spoken by the 3^rd^ male speaker, ‘TF2’ refers to the sound ‘tad’ spoken by the 2^nd^ female speaker, and ‘D90’ refers to the sound ‘dad’ temporally compressed to 90% of the original stimulus length. As in our earlier studies using the same sounds, the speech sounds were shifted up by one octave using the STRAIGHT vocoder [27] in order to better match the rat hearing range [25,28-33]. The intensity of these sounds was adjusted so that the loudest 100 ms of the vowel was 60 dB SPL. Nine temporally compressed versions of ‘dad’ and ‘tad’ spoken by a single female speaker (female 1) were generated using the STRAIGHT vocoder (*n* = 18 sounds). These stimuli were compressed in increments of 10% down to 10% of the original stimulus length. A version of the female 1 ‘dad’ was also created using STRAIGHT with the pitch one octave lower for use during the discrimination training task prior to gender categorization. 

### Behavioral training

Our previous study demonstrated that rats can rapidly learn to discriminate English consonant pairs that differ only in their voicing, place, or manner of articulation [25]. In this study, we tested the ability of rats to categorize sets of 5, 6, 10, or 18 novel sounds based on voicing or gender.

The Voicing Task group (*n* = 6 rats) was trained for two weeks to press a lever in response to ‘dad’ and not to ‘tad’ spoken by female 1. After training, the rats were tested for their ability to correctly categorize eighteen temporally compressed versions of ‘dad’ and ‘tad’. For this go/no-go task, rats were rewarded for responding to any version of ‘dad’ and received a brief time out for false alarming to any version of ‘tad’. Following two weeks of testing on the temporal compression voicing task, the rats were tested for their ability to correctly generalize to ‘dad’ and ‘tad’ produced by five new talkers (2 female and 3 male). 

The Gender Task group (*n* = 5 rats) was trained to lever press in response to ‘dad’ spoken by female 1, but not to the same word when the pitch was shifted down by one octave (F_0_ of 225 Hz) using the STRAIGHT vocoder. After two weeks of pitch discrimination training, the rats were tested for their ability to categorize gender using the novel ‘dad’ stimuli from three male and two female speakers. Rats were rewarded for pressing in response to ‘dad’ spoken by a female, but received a time out for pressing in response to ‘dad’ spoken by a male. Following two weeks of testing on the ‘dad’ gender task, the rats were tested for their ability to correctly categorize gender using the three male and three female ‘tad’ stimuli. Rats were rewarded for lever pressing in response to ‘tad’ spoken by a female, but received a time out for lever pressing in response to ‘tad’ spoken by a male. 

 Training took place in double-walled booths that each contained a speaker (Optimus Bullet Horn Tweeter, Radio Shack), house light, and cage (8” length x 8” width x 8” height) with a lever and pellet dish. The pellet dispenser was mounted outside of the booth to minimize sound contamination. Rats received a 45 mg sugar pellet reward for pressing the lever in response to the target sounds, and received a time out where the house light was extinguished for a period of approximately 6 seconds for pressing the lever in response to the non-target sounds. Rats were food deprived to provide task motivation. Additional food was provided as needed to keep rats between 80% and 90% of their full feed weights. 

 Rats were first trained to press the lever to receive a sugar pellet reward. Each time the rat was near the lever, the rat heard the target sound and received a 45 mg sugar pellet. Pellets were then given only if the rat was touching the lever, and eventually the rat began to press the lever independently. After each lever press, the rat heard the target sound and received a pellet reward. Once they reached the criteria of independently pressing the lever 100 times per session for two sessions, they advanced to the detection phase of training. During this phase, rats from all groups learned to press the lever after hearing the ‘dad’ speech stimulus spoken by female 1. Rats started with an 8 second lever press window (hit window) after each sound presentation, and the hit window was decreased in 0.5 second increments every few sessions as performance increased, down to a hit window of 3 seconds. When rats reached the criteria of a *d*’ ≥ 1.5 for 10 sessions (average of 26 ± 2 sessions), they advanced to the discrimination task. *d*’ is a measure based on signal detection theory of the discriminability of two sets of samples. From this phase on, rats performed each task for 20 sessions over 2 weeks (2 one-hour training sessions per day). Six rats in the Voicing Task group trained on a ‘dad’ vs. ‘tad’ discrimination task for two weeks, followed by a ‘dad’ vs. ‘tad’ temporal compression categorization task for two weeks, followed by a ‘dad’ vs. ‘tad’ multiple speaker categorization task for two weeks. Five rats in the Gender Task group trained on a ‘dad’ pitch discrimination task for two weeks, followed by a ‘dad’ gender categorization task for two weeks, followed by a ‘dad’ and ‘tad’ gender categorization task for two weeks. The final categorization task in each group used the exact same stimuli, ‘dad’ and ‘tad’ spoken by multiple male and female speakers. The Voicing Task group was trained to categorize these stimuli based on voicing, while the Gender Task group was trained to categorize these stimuli based on gender. 

### Anesthetized recordings

 We recorded multi-unit activity (*n* = 441) in the right primary auditory cortex of eleven experimentally naïve female Sprague-Dawley rats in response to each of the 15 ‘dad’ and 15 ‘tad’ stimuli tested behaviorally. Multi-unit recordings were also collected in the right primary auditory cortex of five gender trained rats (*n* = 280 sites) and four voicing trained rats (*n* = 168 sites). Rats were initially anesthetized with pentobarbital (50 mg kg^-1^), and received dilute pentobarbital (8 mg ml^-1^) as needed. Four Parylene-coated tungsten microelectrodes (1–2 MΩ, FHC, Bowdoin, ME, United States) were used to record action potentials ~600 μm below the cortical surface. Recording sites were selected to evenly sample A1 without damaging the cortical surface vasculature.

Each speech sound was presented 20 times (randomly interleaved with a 2 second interstimulus interval). To determine the characteristic frequency of each site, 25 ms tones were presented at 81 frequencies (1 to 32 kHz) and 16 intensities (0 to 75 dB). Stimulus generation, data acquisition, and spike sorting were performed with Tucker-Davis (Alachua, FL, United States) hardware (RP2.1 and RX5) and software (Brainware). Multi-units include action potentials from more than one nearby neuron. The University of Texas at Dallas Institutional Animal Care and Use Committee approved all protocols and recording procedures. 

### Awake recordings

 We recorded multi-unit A1 responses (*n* = 65) in seventeen experimentally naive awake rats using chronically implanted microwire arrays, which were described in detail in previous publications [25,34]. Fourteen-channel microwire electrodes were implanted in the right primary auditory cortex using a custom-built mechanical insertion device to rapidly insert electrodes in layers 4/5 (depth ~600 µm) [34]. Recordings were made in response to the 12 ‘dad’ and ‘tad’ stimuli spoken by 3 male and 3 female speakers, the 18 temporally compressed versions of ‘dad’ and ‘tad’, and the sound ‘dad’ spoken by female 1 with a low pitch (the non-target sound used for discrimination training prior to the gender categorization task). Awake rats were passively exposed to these speech sounds, and were not performing the categorization tasks.

### Data analysis

Neurograms were constructed by arranging the responses from each of the A1 recording sites on the *y* axis from low characteristic frequency to high characteristic frequency sites. The neurogram response for each sound at each site is the average of 20 repeats of that sound played at that site. Neural similarity was computed using Euclidean distance. The Euclidean distance between any two activity patterns is the square root of the sum of the squared differences between the firing rates for each recording site. The onset response to each sound was defined as the 50 ms interval beginning when average neural activity across all sites exceeded the spontaneous firing rate by three standard deviations. Neurograms were temporally binned into a single 50 ms bin. The Euclidean distance was calculated between the activity pattern for a novel sound and both the activity pattern for the target sounds that the rats had previously trained on, and the activity pattern for the non-target sounds that the rats had previously trained on. For the initial gender ‘dad’ categorization task, the previously trained target and non-target template patterns were the response to the high pitch ‘dad’ and low pitch ‘dad’, respectively. For the initial voicing compression task, the template patterns were the response to ‘dad’ and ‘tad’ spoken by female 1. For the second gender task, gender ‘tad’, the template target pattern was the average of the response to the target sounds heard on the previous task (3 female exemplars of ‘dad’), while the template non-target pattern was the average of the response to the non-target sounds heard on the previous task (3 male exemplars of ‘dad’). For the second voicing task, the template target pattern was the average of the response to the target sounds heard on the previous task (10 compressed versions of ‘dad’), while the template non-target pattern was the average of the response to the non-target sounds heard on the previous task (10 compressed versions of ‘tad’). For each novel sound, the distance to the target pattern was subtracted from the distance to the non-target pattern, so that responses with positive values are more similar to the target pattern, while responses with negative values are more similar to the non-target pattern. Pearson's correlation coefficient was used to examine the relationship between neural similarity and generalization performance on the first day of each of the categorization tasks. Our measure of neural similarity is not dependent on the Euclidean distance measure. Neural similarity quantified using City Block distance and Minkowski distance also significantly predicts generalization behavior on all four tasks. To test the importance of spectral precision, neural recordings from 441 A1 sites were binned into subsets containing 1, 2, 3, 4, 5, 7, 9, 10, 15, 20, 25, 55, 110, 220, or 441 sites (441, 220, 147, 110, 88, 63, 49, 44, 29, 22, 17, 8, 4, 2, or 1 bins, respectively). Each bin contained sites tuned to a specific range of frequencies. For example, when the data were divided into four bins, the frequency ranges were 1-6, 6-10, 10-15, and 15-31 kHz.

## Results

### Rats categorize novel speech sounds by speaker gender and voicing

**Figure 2:**
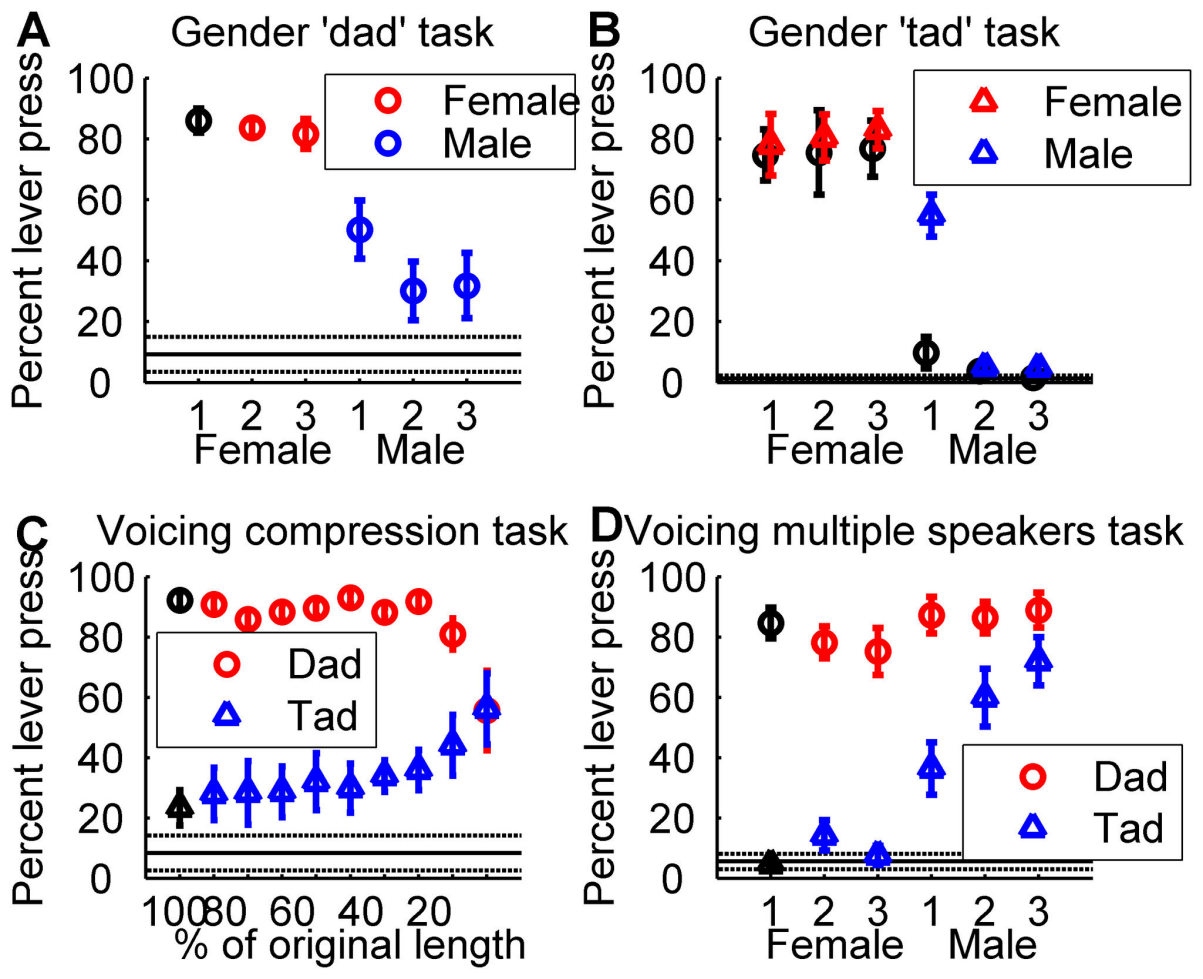
Generalization performance for the gender and voicing categorization tasks. (**a**) Gender Task rats successfully generalized from the pitch discrimination task, and accurately pressed the lever more often in response to novel female ‘dad’ sounds than novel male ‘dad’ sounds on the first day of testing. Red symbols represent target sounds, blue symbols represent non-target sounds, and black symbols represent target or non-target sounds from the previous task. Circle symbols indicate ‘dad’ stimuli, while triangle symbols indicate ‘tad’ stimuli. Error bars indicate s.e.m. across rats. The solid line indicates average percent lever press to silent catch trials, with s.e.m. indicated by the dotted lines. (**b**) Gender Task rats successfully generalized from the gender ‘dad’ categorization task, and accurately pressed the lever more often in response to novel female ‘tad’ sounds than novel male ‘tad’ sounds on the first day of testing. The sounds presented in subplot d are identical. (**c**) Voicing Task rats successfully generalized from the voicing discrimination task, and accurately pressed the lever more often in response to novel temporally compressed ‘dad’ than novel temporally compressed ‘tad’. (**d**) Voicing Task rats successfully generalized from the voicing temporal compression categorization task, and accurately pressed the lever more often in response to ‘dad’ spoken by multiple novel speakers than ‘tad’ spoken by multiple novel speakers.

Rats accurately generalized to novel sounds after training to discriminate a single sound from each of two distinct categories. The Gender Task group of rats (n = 5) was first trained to discriminate the word ‘dad’ with a high pitch from the word ‘dad’ with a low pitch. Following pitch training, the rats were tested on their ability to categorize the gender of novel ‘dad’ sounds spoken by different male and female speakers. Rats were able to perform the task well above chance on the first day of testing (*d*’ = 1.32 ± 0.3 mean ± se, 83 ± 4% lever press to female vs. 37 ± 10% lever press to male, *p* = 0.008, Figure 2a). Following two weeks of training on the ‘dad’ categorization task, the rats were then tested for their ability to generalize to novel ‘tad’ stimuli spoken by the same three male and three female speakers. The Gender Task rats were able to categorize the novel sounds by gender on the first day of testing (80 ± 8% lever press to female sounds vs. 22 ± 3% lever press to male sounds, *d*’ = 1.76 ± 0.2, *p* = 0.001, Figure 2b). These results demonstrate that pitch trained rats were able to accurately categorize speech sounds by gender while ignoring differences in speaker and voicing. 

Another group of rats (Voicing Task, n = 6) was trained to categorize the same sounds but was required to categorize by voicing while ignoring gender (Figure 1). The Voicing Task group initially learned to discriminate the word ‘dad’ from the word ‘tad’, spoken by a single female speaker. These rats were then tested for their ability to categorize these sounds when temporally compressed to create a set of 9 novel ‘dad’ sounds and 9 novel ‘tad’ sounds (with durations 10 - 90% of the original length). The Voicing Task rats were able to generalize to these new stimuli, and accurately categorized 16 of the 18 novel temporally compressed sounds on the first day of training (86 ± 2% lever press to ‘dad’ vs. 34 ± 7% lever press to ‘tad’, *d*’ = 1.53 ± 0.2, *p* = 0.00002, Figure 2c). This same group of rats was next tested for their ability to generalize to novel ‘dad’ and ‘tad’ stimuli spoken by three male and two female speakers. This set of sounds was identical to the sounds presented to the Gender Task rats for their second generalization task. Voicing Task rats accurately categorized the novel sounds by voicing on the first day of testing (83 ± 6% lever press to ‘dad’ vs. 38 ± 6% lever press to ‘tad’, *d*’ = 1.38 ± 0.1, *p* = 0.00008, Figure 2d). These results demonstrate that voicing trained rats were able to generalize to novel stimuli while ignoring significant variation in stimulus duration, speaker, or gender. 

We analyzed the first trial behavioral response to each new sound for each of the four tasks to confirm that categorization behavior recorded on the first day was indeed due to generalization rather than rapid learning. For the first presentation of each sound, rats pressed the lever consistently more often in response to sounds in the target category compared to sounds in the non-target category (average of 72±4% target lever press vs. 44±6% non-target lever press, *p* = 0.0005). These results confirm that rats are able to accurately categorize novel sounds based on experience with as few as one member of each category. 

### Simple acoustic features cannot fully explain gender and voicing categorization by rats

Historically, speech scientists concluded that each speech category is defined by a set of acoustic features such as pitch, formant frequencies, or voice onset time [35,36]. We measured multiple acoustic features for each of the trained sounds (Table 1), and our results confirm that these features are correlated with generalization performance in rats. The pitch (fundamental frequency, F0), first formant peak and second formant peak of each sound are positively correlated with categorization as a female sound by rats (F0: *R* ^2^ = 0.73, *p* = 0.0004; F1: *R* ^2^ = 0.41, *p* = 0.03; F2: *R* ^2^ = 0.35, *p* = 0.04, for both gender tasks). These cues are also correlated with gender judgments by humans [36,37]. 

**Table 1:** Values for the acoustic cues F0, F1, F2, VOT, and burst duration for each sound.

**Sound**	**F0 (Hz)**	**F1 (Hz)**	**F2 (Hz)**	**VOT (msec)**	**Burst duration (msec)**
**DF1**	225	846	2286	19	11.9
**DF2**	241	908	1921	32	16.9
**DF3**	205	995	1970	23	14.3
**DM1**	114	751	1748	9	6.5
**DM2**	116	753	1816	12	7.1
**DM3**	111	798	1760	7	3.9
**TF1**	236	957	1984	79	35.7
**TF2**	260	950	1918	128	66.2
**TF3**	196	917	1820	106	28.6
**TM1**	117	757	1709	49	26.6
**TM2**	118	798	1774	43	10.4
**TM3**	108	850	1808	76	10.4
**D90**	225	846	2286	17.1	10.7
**D80**	225	846	2286	15.2	10.7
**D70**	225	846	2286	13.3	9.9
**D60**	225	846	2286	11.4	8.9
**D50**	225	846	2286	9.5	7.1
**D40**	225	846	2286	7.6	6.3
**D30**	225	846	2286	5.7	6.6
**D20**	225	846	2286	3.8	4.2
**D10**	225	846	2286	1.9	3.6
**T90**	236	957	1984	71.1	33.1
**T80**	236	957	1984	63.2	25.9
**T70**	236	957	1984	55.3	24.9
**T60**	236	957	1984	47.4	18.8
**T50**	236	957	1984	39.5	14.1
**T40**	236	957	1984	31.6	14.3
**T30**	236	957	1984	23.7	11
**T20**	236	957	1984	15.8	8.2
**T10**	236	957	1984	7.9	5.3

The acoustic cues pitch (F0), formant frequencies F1 and F2, VOT, and burst duration were quantified for each sound using Praat [96] and WaveSurfer [97] software. Please note that the values of F0, F1, and F2 that the rats heard were twice the values listed in the table, while VOT and burst duration were unaffected.

Multiple acoustic features are correlated with generalization performance in the Voicing Task rats. Voice onset time (VOT) and burst duration (the duration of the stop consonant release burst) are both correlated with categorization as an unvoiced consonant by rats (VOT *R* ^2^ = 0.6, *p* = 0.0001 voicing compression task; VOT *R* ^2^ = 0.75, *p* = 0.0002 voicing multiple speaker task; Burst duration *R* ^2^ = 0.46, *p* = 0.001 voicing compression task; Burst duration *R* ^2^ = 0.67, *p* = 0.001 voicing multiple speaker task; Table 1). These acoustic cues also predict voicing categorization in humans [35,36]. Previous literature, however, demonstrates that simple acoustic parameters, such VOT and pitch, cannot explain speech perception, especially in difficult listening conditions. Studies in humans and rats have clearly demonstrated that behavioral performance is preserved when background noise or degradation by noise vocoder are used to eliminate VOT, formant, and pitch cues [15,30,31,38,39].

Our behavioral results suggest that the rats do not use a single acoustic feature to accurately categorize sounds by voicing or gender. The behavioral results were inconsistent with the prediction that rats use pitch (fundamental frequency, F0) to discriminate female from male speakers. Rats reliably categorized ‘tad’ spoken by one of the male speakers (TM1) as a sound spoken by a female even though the pitch was 117 Hz. If the rats categorized the sound based on pitch, they would have been expected to respond as if it was one of the male sounds (F0: 114, 116, 111, 118, & 108 Hz) and not as if it was one of the female sounds (F0: 225, 241, 205, 236, 260, & 196 Hz).

The behavioral results were inconsistent with the prediction that rats use VOT to discriminate ‘dad’ from ‘tad’. Previous studies have shown that humans and rodents categorize sounds as voiced when they have a VOT of less than 35 ms [9]. After our rats were trained to lever press to ‘dad’ (VOT = 19 ms) and not to ‘tad’ (VOT = 79 ms), the rats were tested on versions of these sounds that were temporally compressed such that their VOTs were shortened to 10 to 90% of their initial durations (i.e. 2 to 71 ms, see Table 1). Rats accurately rejected compressed forms of ‘tad’ even when the VOT was much lower than 35 ms (T20 – T40 in Table 1). Importantly, the rats continued to accurately reject compressed ‘tad’ sounds even when the VOT was below the value for the trained target ‘dad’ sound (19 ms). These behavioral responses occurred on the first presentation of these novel sounds, which proves that the categorical boundary was not shifted by experience. If the rats categorized the sounds based on a single acoustic feature, it would be expected that they would respond (i.e. lever press) to any stimulus with a VOT less than 20 ms. However, the rats failed to respond to the ‘tad’ with a VOT of 16 ms (because it was compressed to 20% of the original duration). The fact that the rats continued to reliably press to the 19 ms ‘dad’ demonstrates that they do not categorize the sounds based on a simple measure of acoustic similarity, such as VOT. 

The single acoustic features analyzed here (for example, F0 or VOT) cannot fully explain the response errors; however, combinations of acoustic features may have the potential of accounting for the behavioral results [40]. Based on our previous findings that the similarity of speech evoked spatiotemporal activity patterns was correlated with discrimination ability [25,26,30,31], we predicted that neural similarity would be able to explain generalization behavior. Neural similarity provides a single, biologically plausible method to explain speech sound categorization without the need to propose multiple specialized features for each speech contrast. 

### Neural activity pattern similarity explains gender and voicing categorization

**Figure 3:**
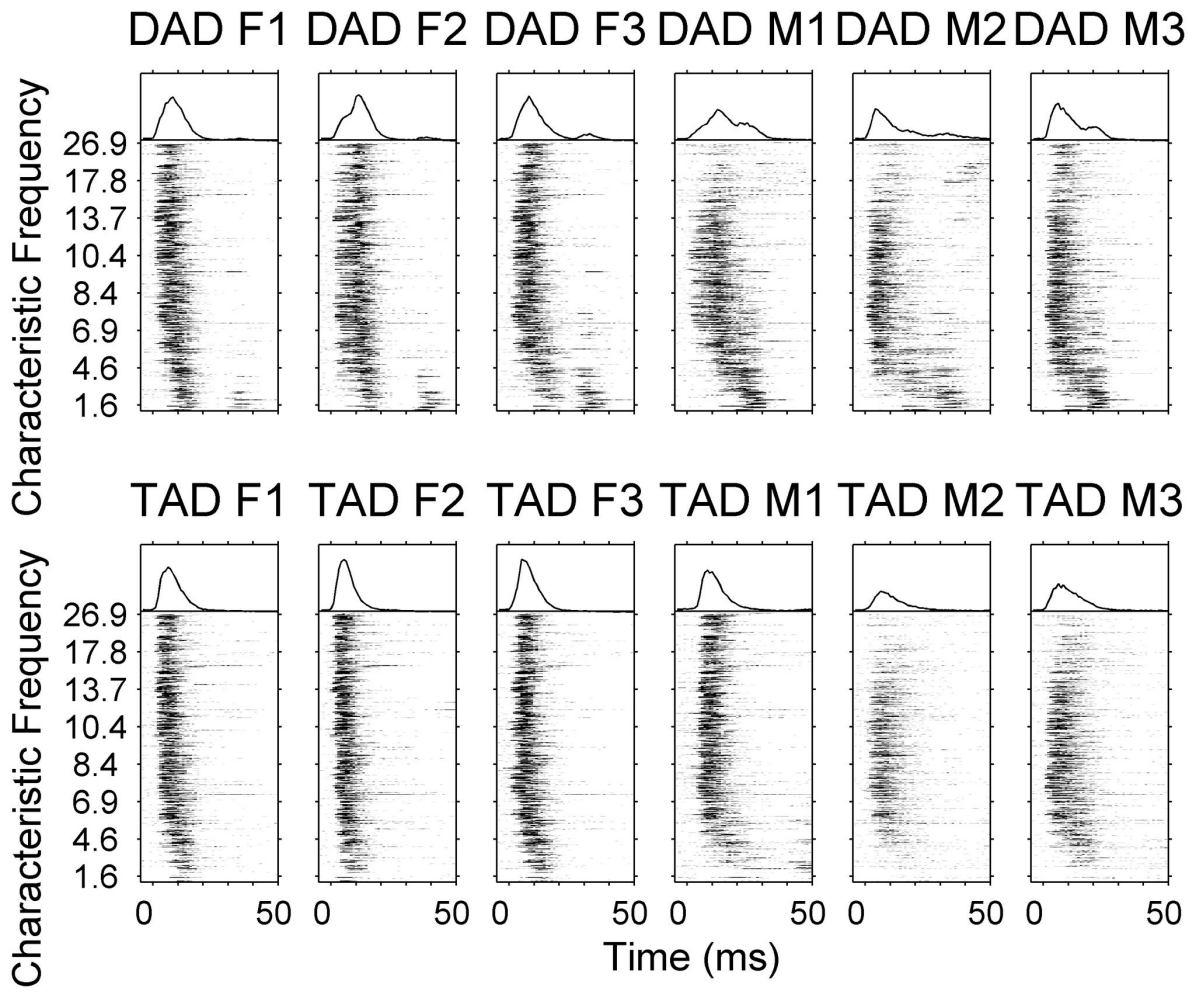
Neurograms depicting the onset response of rat A1 neurons to speech sounds. Multi-unit data was collected from 441 recording sites in eleven anesthetized experimentally naïve adult rats. Average post-stimulus time histograms (PSTH) derived from twenty repeats were ordered by the characteristic frequency (kHz) of each recording site (*y* axis). Time is represented on the *x* axis (-5 to 50 ms). The firing rate of each site is represented in grayscale, where black indicates 450 spikes/s. For comparison, the mean population PSTH evoked by each sound is plotted above the corresponding neurogram. To facilitate comparison between the naïve and trained responses, the mean PSTH y axis is set to 450 Hz for all neurogram figures. For naïve rats, ‘tad’ female #3 evokes the maximum peak firing rate (351 Hz) across the twelve sounds. As in Figure 1, rows differ in voicing (top row is ‘dad’, bottom row is ‘tad’), while columns differ in gender (left three columns are female, right three columns are male).

We predicted that rats would compare the neural pattern of activity evoked by each novel sound with stored templates of the target and non-target sounds. Rats made generalization errors more often for some sounds than others, and these errors were well explained by comparing the activity pattern for those sounds with the template patterns. For each task, neural similarity was calculated between the activity pattern for a novel sound and the average activity patterns for the target and non-target sounds from the previous task (Figure 3, Figure S1, and Methods). For the first gender generalization task, rats had previously been trained to discriminate the word ‘dad’ with a high pitch from the word ‘dad’ with a low pitch. Since the rats only had experience with the two ‘dad’ sounds, the stored target template for the first gender generalization task was the activity pattern in response to the word ‘dad’ with a high pitch, and the stored non-target template was the activity pattern in response to the word ‘dad’ with a low pitch. For the second gender generalization task, the rats had experience with ‘dad’ spoken by 3 female speakers and 3 male speakers. The stored target template for the second gender generalization task was the average activity pattern in response to ‘dad’ spoken by the 3 female speakers, while the stored non-target template was the average activity pattern in response to ‘dad’ spoken by the 3 male speakers.

**Figure 4:**
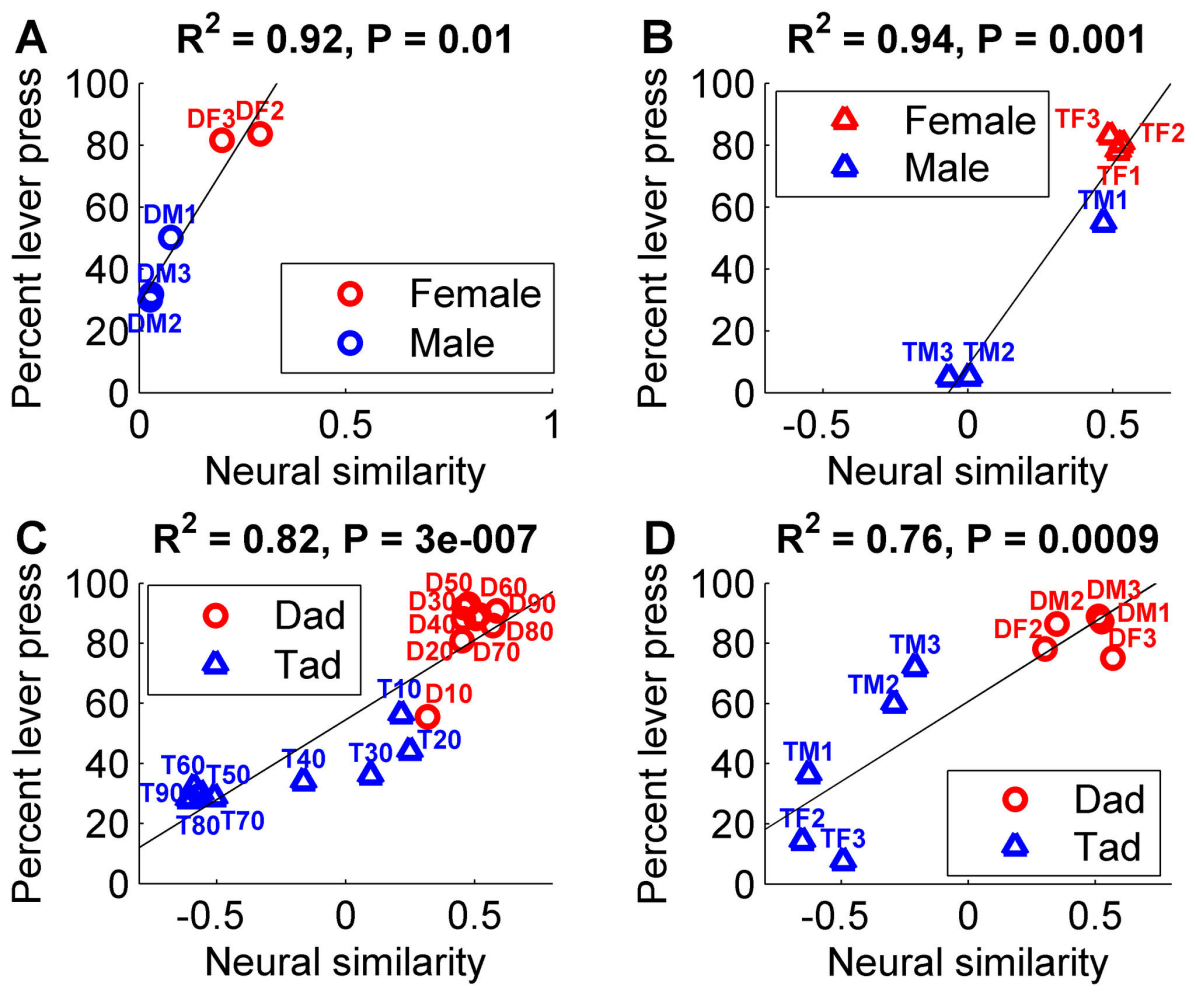
Neural correlates of generalization performance for the gender and voicing categorization tasks. (**a**) The normalized Euclidean distance (neural similarity) between the response pattern for each novel sound and the response pattern for each of the two template sounds is correlated with generalization performance on the gender ‘dad’ task. Positive values are more similar to the target template, while negative values are more similar to the non-target template. Target sounds are red, and non-target sounds are blue. The sound name abbreviation is printed next to each data point, see Methods. Solid lines indicate the best linear fit. (**b**) The neural similarity between each novel sound and the template sounds is correlated with generalization performance on the gender ‘tad’ task. (**c**) The neural similarity between the response pattern for each novel sound and the response pattern for each of the two template sounds is correlated with generalization performance on the voicing temporal compression categorization task. (**d**) The neural similarity between each novel sound and the template sounds is correlated with generalization performance on the voicing multiple speaker task.

The pattern of generalization errors on the gender tasks was well explained by the similarity of the activity patterns evoked by each of the novel sounds to the patterns evoked by each of the trained sounds. As we predicted, rats were most likely to make generalization errors in response to the novel sounds which evoked neural activity patterns that were intermediate between the patterns evoked by the target and non-target sounds. We used a Euclidean distance metric to quantify the similarity of primary auditory cortex responses. Response patterns consisted of the onset response from 441 multiunit A1 sites from 11 anesthetized experimentally naive rats. As predicted, neural similarity between the novel sound and the trained sounds was strongly correlated with generalization performance for both gender generalization tasks (*R* ^2^ = 0.92, *p* = 0.009 novel ‘dad’ sounds; *R* ^2^ = 0.94, *p* = 0.001 novel ‘tad’ sounds, Figures 3 & 4a,b). These findings support our hypothesis that neural similarity provides a biologically plausible metric of perceptual similarity.

Generalization errors were well explained by comparing the neural response pattern evoked by each of the novel sounds to the patterns evoked by the trained sounds. For example, during the second gender task, rats frequently incorrectly pressed the lever for the ‘tad’ spoken by male 1 (TM1, Figure 4b). Based solely on the acoustic feature pitch, the rats should have responded as though the sound was male instead of female (see Acoustic features section above). This error is well explained using neural similarity, where the sound more closely resembles the female template compared to the male template (Figure 4b). By examining the neurogram for this sound (Figure 3), it is clear that the sound evokes a strong high frequency response, which makes the response more closely resemble the female sounds (which also evoke a strong high frequency response) compared to the other male sounds (which evoke a weak high frequency response). 

The pattern of generalization errors on the voicing tasks was well explained by the similarity of the activity patterns evoked by each of the novel sounds to the patterns evoked by each of the trained sounds. As we predicted, neural similarity between the novel sound and the trained sounds was correlated with generalization performance for both voicing generalization tasks (*R* ^2^ = 0.82, *p* < 0.0001 voicing compression task; *R* ^2^ = 0.76, *p* = 0.0009 voicing multiple speaker task, Figures 3 & 4c,d). As seen for the gender tasks, generalization errors on the voicing tasks were well explained by the neural responses. Rats frequently incorrectly responded to the most compressed versions of ‘tad’ as though they were ‘dad’ (Figure 4c). The generalization errors to the most compressed versions of ‘tad’ can be explained by neural responses but are not well explained by the acoustic feature voice onset time. Results from the Voicing Task group confirm our hypothesis that novel speech sounds are assigned to the speech category whose members generate an average activity pattern that most closely resembles the activity pattern evoked by the novel sound. This finding is consistent with earlier predictions that have never been tested. In the natural world, humans and animals generally have experience with more than one exemplar per category. The similarity-based prototype model proposes that a category prototype is the most typical member of the category [41]. An extension of this model proposes that instead of category prototypes being the best examples from their categories, prototypes are an abstraction composed of the average category member [42]. As we predicted, rats with previous categorization experience appear to store templates of the target and non-target sounds based on the average neural responses evoked by the sounds they have experienced, and compare the pattern of activity evoked by each novel sound in the new task to these stored average templates. 

### Generalization performance is not well correlated with spectrogram similarity

**Figure 5:**
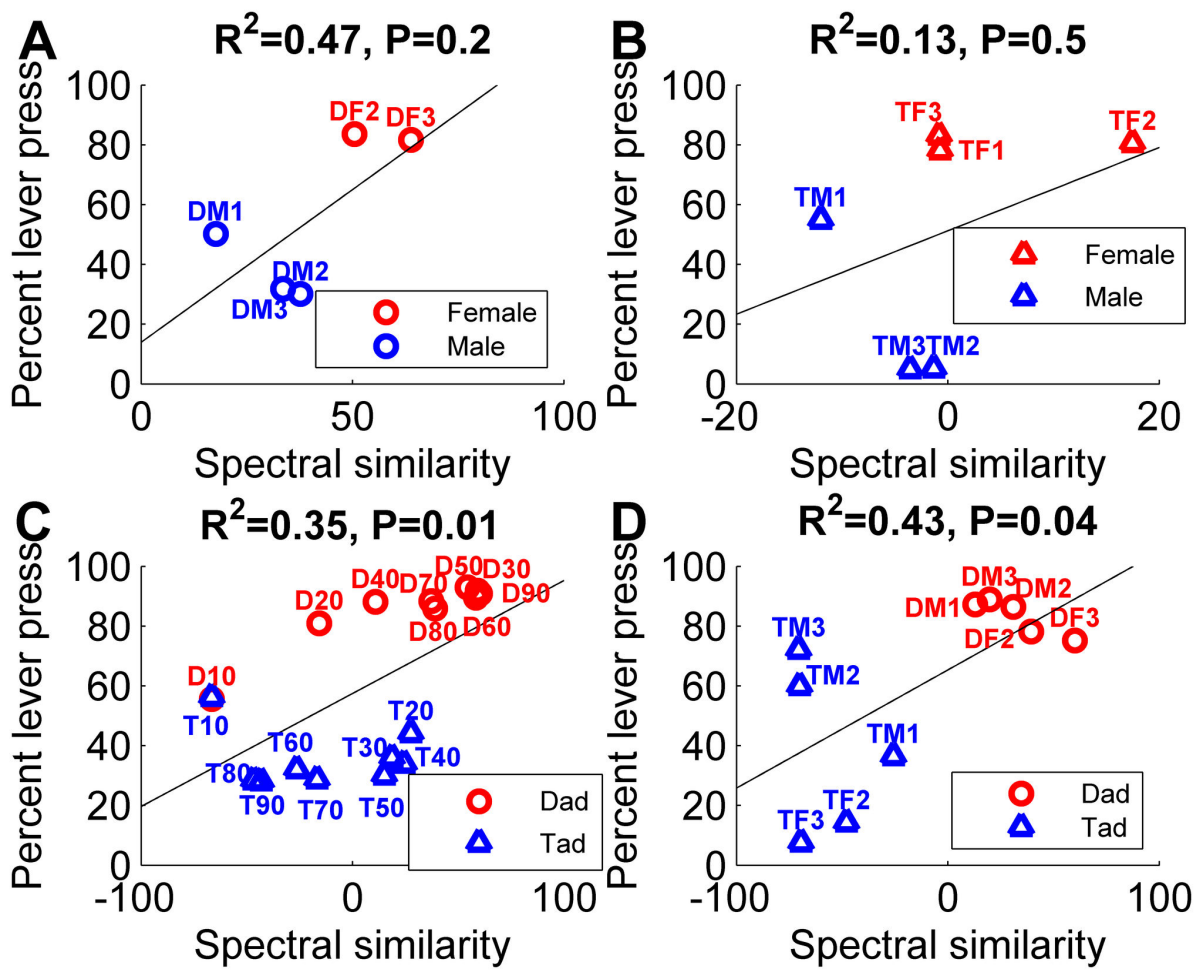
Spectrogram correlates of generalization performance for the gender and voicing categorization tasks. (**a**) The Euclidean distance (spectral similarity) between the spectrogram for each novel sound and the spectrogram for each of the two template sounds is weakly correlated with generalization performance on the gender ‘dad’ task. Positive values are more similar to the target template, while negative values are more similar to the non-target template. The sound name abbreviation is printed next to each data point, see Methods. Solid lines indicate the best linear fit. (**b**) The spectral similarity between each novel sound and the template sounds is weakly correlated with generalization performance on the gender ‘tad’ task. (**c**) The spectral similarity between the spectrogram for each novel sound and the spectrogram for each of the two template sounds is weakly correlated with generalization performance on the voicing temporal compression categorization task. (**d**) The spectral similarity between each novel sound and the template sounds is weakly correlated with generalization performance on the voicing multiple speaker task.

For each task, spectrogram similarity was calculated between the power spectrum for a novel sound and the average power spectrums for the target and non-target sounds from the previous task. The Euclidean distances between the spectrograms of the speech onsets (45 ms) were only moderately correlated with behavior (*R* ^2^ = 0.35, *p* = 0.01 voicing compression task; *R* ^2^ = 0.43, *p* = 0.04 voicing multiple speaker task; *R* ^2^ = 0.47, *p* = 0.20 gender ‘dad’ task; *R* ^2^ = 0.13, *p* = 0.48 gender ‘tad’ task; Figure 5). The first 45 ms of the spectrograms were used to match the 50 ms neural analysis window, excluding 5 ms to account for minimum neural delay. A similar pattern of correlation was observed across a wide range of analysis windows. Analysis of the onset power spectrum alone is not able to accurately predict generalization behavior for the four tasks because spectral analysis is only influenced by spectral energy and does not take into account the temporal characteristics of the acoustic energy or the neural response properties. Thus, it is perhaps not surprising that neural analysis more accurately predicts behavior. 

### Responses from trained rats are correlated with generalization performance

Previous studies have documented primary sensory cortex plasticity following categorization training [43]. We tested whether voicing and gender categorization training led to long lasting changes in A1 responses and if so, whether these changes would be expected to improve categorization. After two weeks of training, performance on each task was significantly better than first day performance (two-way ANOVA, *F*_1,39_ = 10.31, *p* = 0.0026). Across all tasks, rats categorized the speech sounds 34% more accurately on the last day compared to the first day (last day *d*’ = 2.08 ± 0.27 vs. first day *d*’ = 1.55 ± 0.09). Several studies have suggested that enhanced neural responses are responsible for improved categorization [43-45]. The response strength to the trained sounds (i.e. ‘dad’ and ‘tad’ spoken by female and male speakers) in both trained groups did not increase compared to naïve controls (2.5 ± 0.2 spikes in trained rats vs. 2.5 ± 0.2 spikes in naïve rats, *p* = 0.97). The response strength to untrained speech sounds (‘pad’, ‘kad’, ‘zad’, ‘wad’, ‘had’) was also not increased in trained rats compared to naïve control rats (1.9 ± 0.3 spikes in trained rats vs. 1.7 ± 0.2 spikes in naïve rats, *p* = 0.53), but the response strength to tones was decreased in trained rats (2.1 ± 0.1 spikes in trained rats vs. 3 ± 0.3 spikes in naïve rats, *p* = 0.005). The onset latency to the trained sounds in both trained groups did not change compared to naïve controls (11.1 ± 0.7 ms in trained rats vs. 11.5 ± 0.6 ms in naïve rats, *p* = 0.64). 

While these results show that categorization training does not enhance auditory cortex response strength, it does not rule out that plasticity plays a role in generalization performance. To determine if auditory cortex plasticity enhanced the distinction between sounds from different categories, we compared the correlation between neural similarity and generalization performance using neural responses collected from voicing and gender trained rats (Figure S2). If auditory cortex plasticity is required in order to accurately predict performance, we would have expected a stronger correlation between neural similarity and generalization performance using neural responses collected from trained compared to naïve rats. The neural similarity between the novel sound and template sound responses collected in trained rats predicted generalization performance on both gender categorization tasks (*R* ^2^ = 0.91, *p* = 0.01, gender ‘dad’ task; *R* ^2^ = 0.96, *p* = 0.0006, gender ‘tad’ task; Figure S3a,b) and both voicing categorization tasks (*R* ^2^ = 0.71, *p* < 0.0001, voicing compression task; *R* ^2^ = 0.58, *p* = 0.01, voicing multiple speaker task; Figure S3c,d). Neural similarity was highly correlated with generalization performance on each of the four tasks whether the neural responses were recorded in naïve or trained rats (naïve average *R* ^2^ = 0.86, *p* < 0.01; trained average *R* ^2^ = 0.79, *p* < 0.02). This result is consistent with earlier reports that speech sounds evoke distinct neural patterns before training begins [25,26,30,31,46]. The average Euclidean distance between stimuli from different categories was not increased in trained rats compared to naïve rats (*p* > 0.05). Our observation suggests that changes in A1 are not responsible for improved performance (see Text S1). Previous studies have detailed the complexity of training-induced plasticity, which is dependent on both the auditory field and the time course of training. Birds trained to discriminate songs have shown either an increase or a decrease in the response strength to familiar songs compared to unfamiliar songs depending on the auditory field [44,47]. Earlier studies have also reported improved categorization in the absence of plasticity in primary sensory cortex [48-52]. Training induced map plasticity in A1 can later return to a normal topography without negatively impacting behavioral performance [49,53]. Improved performance may result from changes in higher cortical fields, such as the superior temporal gyrus or prefrontal cortex, that exhibit categorical responses to speech sounds [54-57].

### Analysis of categorization by different neural subpopulations

**Figure 6:**
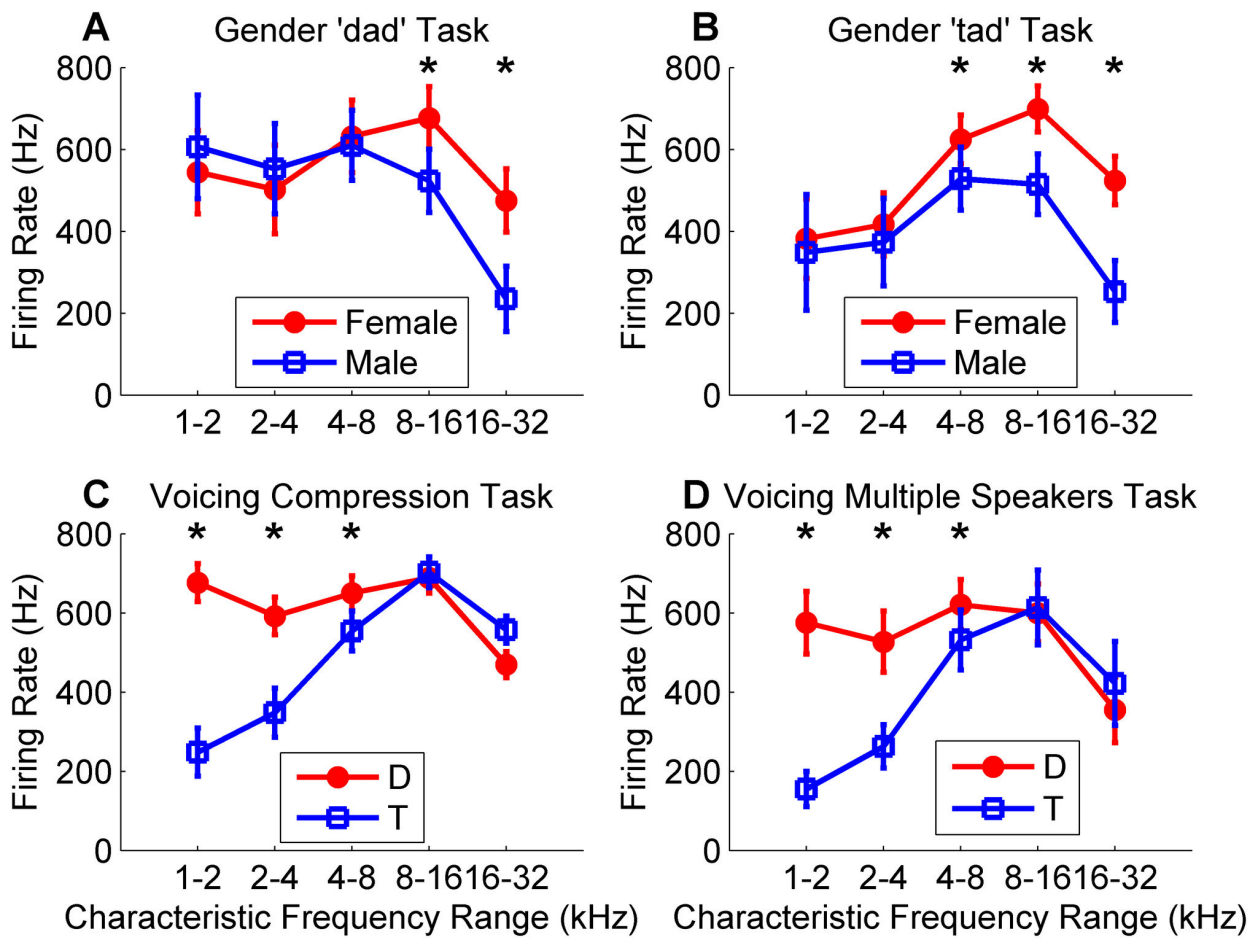
Peak firing rate differences in high and low frequency neurons for gender and voicing distinctions. Peak firing rate for target and non-target sounds differs in high frequency neurons for gender distinctions, and differs in low frequency neurons for voicing distinctions. **(a)** For the gender task using ‘dad’ stimuli, target female ‘dad’ sounds evoke a larger response in high frequency neurons compared to non-target male ‘dad’ sounds. Each of the 441 A1 recording sites from experimentally naïve rats were binned by characteristic frequency into one of five bins each spanning one octave. Error bars indicate s.e.m. across each of the sounds. **(b)** For the gender task using ‘tad’ stimuli, target female ‘tad’ sounds evoke a larger response in high frequency neurons compared to non-target male ‘tad’ sounds. **(c)** For the voicing temporal compression task, target ‘dad’ sounds evoke a larger response in low frequency neurons compared to non-target ‘tad’ sounds. **(d)** For the voicing multiple speaker task, target ‘dad’ sounds evoke a larger response in low frequency neurons compared to non-target ‘tad’ sounds.

The patterns of neural activity evoked by each of the sounds suggest that gender differences are encoded in the onset response of high frequency neurons, while voicing differences are encoded in the onset response of low frequency neurons (Figures 3, 6 and S4). For the gender tasks, sounds spoken by a female evoked 207% more spikes than sounds spoken by a male in high frequency neurons between 16 and 32 kHz (*p* < 0.0001, Figure 6a,b), but there was no significant difference in the firing rate in low frequency neurons between 1 and 2 kHz (*p* = 0.66). In contrast to gender firing differences, ‘dad’ sounds evoked 302% more spikes than ‘tad’ sounds in low frequency neurons between 1 and 2 kHz (*p* < 0.0001, Figure 6c,d), but there was a much smaller difference in the firing rate in high frequency neurons between 16 and 32 kHz (16% fewer spikes, *p* = 0.05). This finding contrasts with earlier reports suggesting that voicing is encoded in the temporal interval between two activity peaks [22], and pitch is encoded in low frequency neurons [21]. Our results suggest that the spatial activity pattern can be used to accurately categorize these speech sounds. 

**Figure 7:**
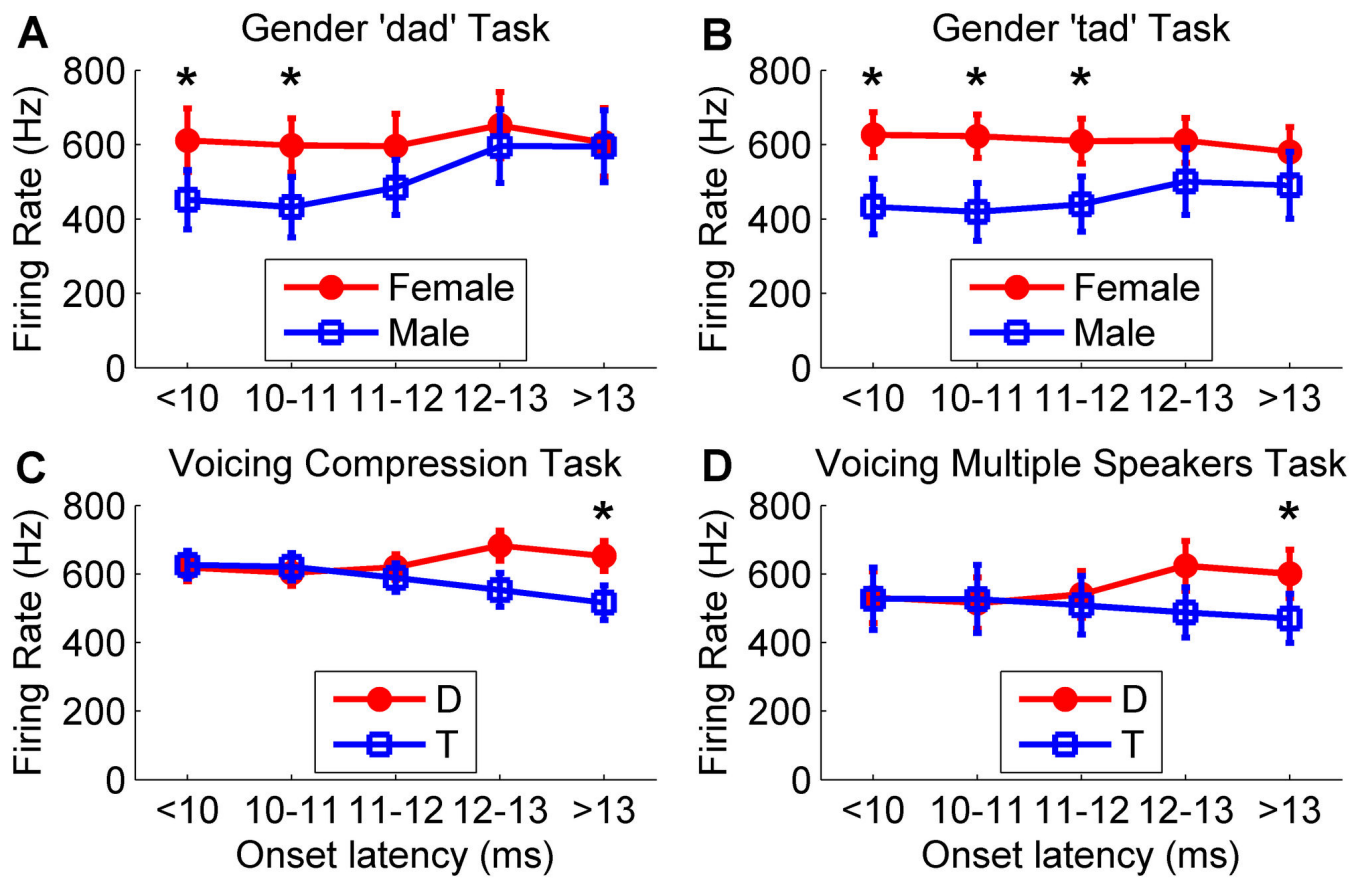
Peak firing rate differences in fast and slow latency neurons for gender and voicing distinctions. Peak firing rate for target and non-target sounds differs in fast neurons for gender distinctions, and differs in slow neurons for voicing distinctions. (**a**) For the gender task using ‘dad’ stimuli, target female ‘dad’ sounds evoke a larger response in fast neurons that respond to tones in less than 10 ms compared to non-target male ‘dad’ sounds. Each of the 441 A1 recording sites from experimentally naïve rats were binned by onset latency into one of five bins each spanning one millisecond. Error bars indicate s.e.m. across each of the sounds. (**b**) For the gender task using ‘tad’ stimuli, target female ‘tad’ sounds evoke a larger response in fast neurons compared to non-target male ‘tad’ sounds. (**c**) For the voicing temporal compression task, target ‘dad’ sounds evoke a larger response in slow neurons that respond to tone slower than 13 ms compared to non-target ‘tad’ sounds. (**d**) For the voicing multiple speaker task, target ‘dad’ sounds evoke a larger response in slow neurons compared to non-target ‘tad’ sounds.

The temporal activity pattern also contains information that can be used to accurately categorize the sounds by voicing or gender. For the gender tasks, sounds spoken by a female evoked 45% more spikes than sounds spoken by a male in neurons responding to a tone faster than 10 ms (< 0.0001, Figure 7a,b and Figure S5), but there was no significant difference in the firing rate in neurons responding slower than 13 ms. In contrast to gender firing differences, ‘dad’ sounds evoked 28% more spikes than ‘tad’ sounds in neurons responding to a tone slower than 13 ms (*p* = 0.001, Figure 7c,d and Figure S5), but there was no significant difference in the firing rate in neurons responding faster than 10 ms. Our results suggest that both the spatial and the temporal activity pattern can be used to accurately categorize these speech sounds. 

There are many potential methods to compute the similarity between neural response patterns that accurately predict generalization performance. Neural similarity was highly correlated with generalization performance for all four tasks whether Euclidean, City Block, or Minkowski distance metrics were used (*R* ^2^ > 0.73, *p* < 0.03). The correlation remains high if the window used to quantify the neural response ends 30 to 120 ms after sound onset (*R* ^2^ > 0.51, *p* < 0.03, Figure S6a and Figure S7). Neural similarity is only correlated with generalization performance when the onset response is included in the analysis window (*p* < 0.05, Figure S6b). This finding is consistent with classic studies showing speech sounds can be accurately categorized using only the initial few tens of milliseconds [58,59]. Although our initial analyses considered the neural responses of each A1 recording site separately, to determine the amount of spectral precision that is necessary, we divided sites into bins that were tuned to specific characteristic frequency ranges. The correlation between generalization performance and neural similarity remains high even if the sites are binned by characteristic frequency into as few as two bins (*R* ^2^ > 0.61, *p* < 0.01) [30]. The consistency of our results across a wide range of parameters supports our hypothesis that the similarity to previously learned patterns is used to categorize novel stimuli. These results are consistent with recent imaging results that even neural metrics with poor spatial and temporal precision can be well correlated with categorization performance [60,61]. 

**Figure 8:**
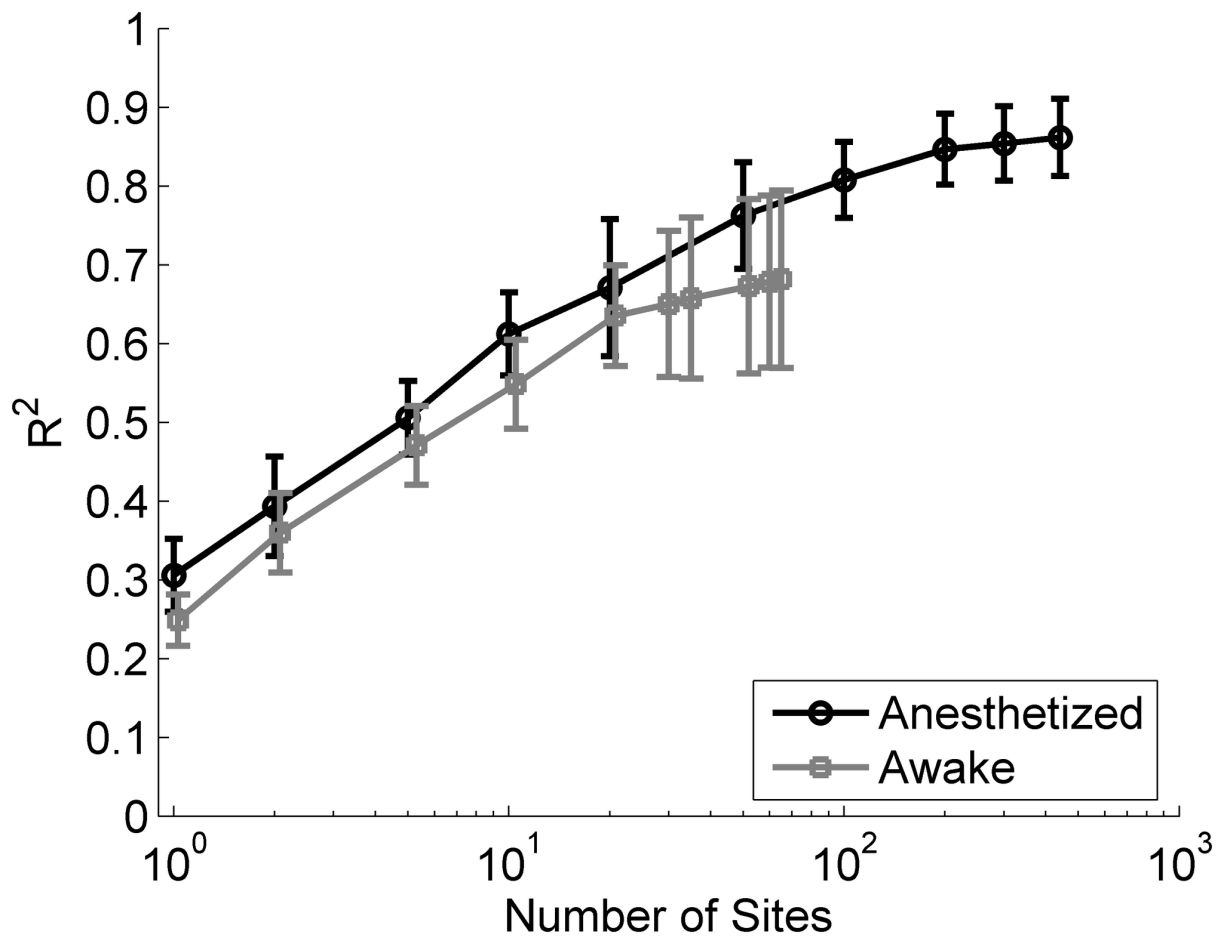
Average percent of variance explained across the four generalization tasks using awake and anesthetized responses. Percent of variance explained (R^2^) increases as the population size increases. Neural similarity using the onset activity pattern from individual anesthetized (black line) or awake (gray line) multi-unit sites was best correlated with behavior when more than 20 sites were used. Error bars indicate s.e.m. across the four tasks.

Neural similarity accurately predicts generalization performance using both awake and anesthetized neural responses. The correlation between neural similarity and generalization performance using neural responses from experimentally naïve awake rats was strong for the gender ‘dad’ task (*R* ^2^ = 0.89, *p* = 0.02), the voicing temporal compression task (*R* ^2^ = 0.61, *p* = 0.0001), the gender ‘tad’ task (*R* ^2^ = 0.78, *p* = 0.02), and the voicing multiple speaker task (*R* ^2^ = 0.44, *p* = 0.04). This result strengthens our finding in experimentally naïve anesthetized rats that auditory cortex plasticity is not required to predict generalization performance. Using both anesthetized and awake responses, we examined how large of a neural population must be sampled to accurately estimate neural similarity and generalization performance. Given the great diversity of response properties in A1 [62], we expected that a large sample size might be necessary. We randomly selected groups of 1, 2, 5, 10, 20, 50, 100, 200, 300, or 441 anesthetized A1 sites and randomly selected groups of 1, 2, 5, 10, 20, 30, 35, 50, 60, or 65 awake A1 sites to determine the minimum population size required in order to predict generalization. We found that the correlation between neural similarity and generalization performance becomes significant when more than 20 randomly selected multi-unit clusters were used to estimate each neural activity pattern and asymptotes at approximately 100 (*p* < 0.05, Figure 8). 

Neural similarity was not better correlated with generalization behavior when A1 neurons were selected to maximize the difference in the evoked responses. Selecting subpopulations also did not reduce the number of A1 sites needed to generate a significant correlation. For example, when A1 sites with low frequency tuning (< 8 kHz, Figure 6) and long latency (>13 ms, Figure 7) were used to compute neural similarity and compared with performance on the voicing task, approximately the same number of sites were required to generate a similar correlation coefficient compared to neural similarity based on a randomly selected set of A1 sites. The consequence was the same when subpopulations were used that generated the maximum response difference for the gender task (i.e. high frequencies and short latencies). These results confirm earlier observations that population responses most accurately reflect behavioral ability [25,63]. There is now strong evidence that the degree of abstraction increases with distance from the receptor surface (e.g. cochlea) and that categorization is the result of neural processing distributed across many brain regions [64].

## Discussion

We tested the hypothesis that the similarity between neural activity patterns predicts speech sound generalization without the need to compute multiple acoustic features. Speech sounds are widely believed to be categorized based on the integration of dozens of acoustic features. At least sixteen features have been proposed to contribute to differences in voicing, including voice onset time, pitch contour, burst intensity, and F1 cutback [11]. Separate sets of acoustic features can be used to distinguish between speech sounds differing in gender, place of articulation, vowel, or frication [12,37,65-69]. While any of these features is sufficient to categorize a speech sound, no particular acoustic difference is required to accurately categorize a sound [13,14]. Our results from four voicing or gender speech categorization tasks suggest that template matching in the brain can account for the classic “lack of invariance” of speech perception without requiring storage and analysis of the relationship between a large number of discrete features. Our study failed to find evidence of neurons tuned exclusively to one acoustic feature of speech sound (i.e. VOT or pitch). This result is consistent with a recent study demonstrating that responses in auditory cortex neurons can be influenced by multiple acoustic features of speech sounds [70]. 

The behavioral and physiological results from our study confirm and extend findings from earlier studies [40]. Our observation that rats trained to discriminate sounds based on voicing or gender can accurately categorize novel sounds even on the first presentation confirms previous studies showing that animals can categorize sounds based on voicing or gender differences [9,51,71]. The neural responses collected in this study are similar to earlier reports of speech sound responses in humans and animals [22,51,72,73]. 

Our demonstration that speech perception can be explained without explicit extraction of specialized acoustic features closely parallels recent advances in face processing which no longer relies on the computation of features such as pupil to pupil distance, nose length, or chin shape. Instead it appears that biological systems and more effective artificial systems represent the visual input as activity among a large diverse set of broadly tuned filters and categorize novel inputs based on their similarity to stored templates. Importantly, there is no need to extract any particular features. Recent software applications use a similar featureless template-based method to allow for identification of millions of songs based on poor quality versions sung, whistled, hummed, or played by amateurs [19,20,74]. 

Our results are consistent with other studies of category formation in other modalities [57,75-80]. Previous studies have shown that there is a gradual transformation of sensory information to a category decision through the ascending somatosensory, visual, and auditory pathways [75,76,81]. The earlier stages of sensory processing are driven by physical properties. Responses in primary sensory cortex are more abstract and are often shaped by multiple feature combinations [82-85]. Higher cortical fields are shaped by behavioral requirements and neurons become more sensitive to the meaning of stimuli and less sensitive to changes in physical characteristics that are irrelevant to category membership. Neurons in prefrontal cortex exhibit strong category selectivity and likely contribute to the behavioral response (i.e. motor output) [8,76,86,87]. 

Speech responses in inferior colliculus are strongly influenced by physical features, while responses in A1 are more abstract [28,60,79]. Responses in higher auditory fields follow different processing streams that extract different features from speech [32,53,81,88-90]. For example, anterior auditory field is responsible for categorization based on temporal properties and posterior auditory field is responsible for categorization based on spatial location [88]. Macaques trained to discriminate between human speech sounds have neurons in the superior temporal gyrus and prefrontal cortex that respond categorically to the trained sounds [56,57]. Prefrontal neurons (but not the superior temporal gyrus neurons) are modulated by the monkeys' behavioral responses, which confirms that speech categories result from the gradual transformation of acoustic information across multiple brain regions. 

 We do not believe that categorization takes place in A1. Our results are consistent with earlier theoretical studies showing that categorical responses can be created from the activity patterns observed in sensory cortex [54,91-93]. For example, a biologically plausible model of A1 neurons can categorize speech sounds and correctly generalize to novel stimuli [54]. These theoretical studies combined with our neurophysiology study suggest a potential biological mechanism for generalization, which has been described as “the most fundamental problem confronting learning theory” [94].

Based on our observation that neural similarity can accurately predict categorization on four auditory generalization tasks, we propose that speech sound generalization results from assigning novel stimuli to the category of stimuli that evokes the most similar activity pattern. Animal studies provide the opportunity to carefully control the sensory experience of the subjects and to precisely manipulate neural function. Artificial stimuli produced by the Klatt speech synthesizer could be used to explore the co-variation between the acoustic features which were not varied systematically in this study [12]. It would be interesting to determine how well neural similarity predicts generalization behavior 1) in the face of greater variability among stimuli from the same category, 2) for categories of stimuli involving multiple modalities, and 3) for more abstract cognitive categories. It would also be interesting to relate behavioral reaction time and neural similarity by using nose poke withdrawal to more accurately measure reaction time. Patterned optogenetic stimulation could be used to directly test whether the activity patterns observed in our study are sufficient for speech sound categorization [95]. Simultaneous multichannel recordings in awake behaving animals would make it possible to relate neural correlation patterns to behavior. Recording, lesion and microstimulation experiments in A1 and higher regions are needed to further evaluate our hypothesis that neural response similarity is responsible for the remarkable ability of humans and animals to rapidly and accurately generalize from small training sets.

## Supporting Information

Figure S1 **Neural similarity between two novel sounds and the trained target and trained non-target.** Multi-unit data was collected from 441 recording sites (*x* axis) in eleven anesthetized rats and is ordered by the characteristic frequency (kHz) of each recording site. The number of spikes fired in response to each sound during the first 50 ms of the response is represented on the *y* axis. (**a**) The response to the known target sound (red, ‘dad’ spoken by female #1) and (**b**) known non-target sound (blue, ‘tad’ spoken by female #1). (**c**) The response to a novel ‘dad’ sound and a novel ‘tad’ sound (**d**). Both sounds were spoken by female #1 and temporally compressed by 50%. (**e**-**h**) The response pattern difference between the novel ‘dad’ sound and the target (**e**) and non-target sounds (**f**), and the novel ‘tad’ sound and the target (**g**) and non-target sounds (**h**). The difference between the novel ‘dad’ and the target (e, 309) was smaller than the difference between the novel ‘dad’ and the non-target (f, 536), indicating that the novel ‘dad’ and the target are more similar. The difference between the novel ‘tad’ and the non-target (h, 267) was smaller than the difference between the novel ‘tad’ and the target (g, 535), indicating that the novel ‘tad’ and the non-target are more similar.
(PDF)

Figure S2 **Neurograms depicting the onset response of gender trained and voicing trained rat A1 neurons.** (**a**) Multi-unit data was collected from 280 recording sites in five anesthetized gender trained rats. Average post-stimulus time histograms (PSTH) derived from twenty repeats were ordered by the characteristic frequency (kHz) of each recording site (*y* axis). Time is represented on the *x* axis (-5 to 50 ms). The firing rate of each site is represented in grayscale, where black indicates 450 spikes/s. For comparison, the mean population PSTH evoked by each sound is plotted above the corresponding neurogram. To facilitate comparison between the naïve and trained responses, the mean PSTH y axis is set to 450 Hz for all neurogram figures. For gender trained rats, ‘tad’ female #3 evokes the maximum peak firing rate (330 Hz) across the twelve sounds. As in Figure 1, rows differ in voicing (top row is ‘dad’, bottom row is ‘tad’), while columns differ in gender (left three columns are female, right three columns are male). (**b**) Neurograms depicting the onset response of voicing trained rat A1 neurons to each of the twelve sounds shown in Figure 1. Multi-unit data was collected from 168 recording sites in four anesthetized voicing trained rats. For voicing trained rats, ‘tad’ female #2 evokes the maximum peak firing rate (414 Hz) across the twelve sounds.
(PDF)

Figure S3 **Neural correlates of generalization performance using neural responses from gender and voicing trained rats.** (**a**) The normalized Euclidean distance (neural similarity) between the response pattern for each novel sound and the response pattern for each of the two template sounds is correlated with generalization performance on the gender ‘dad’ task. Positive values are more similar to the target template, while negative values are more similar to the non-target template. Red symbols represent target sounds and blue symbols represent non-target sounds. Circle symbols indicate ‘dad’ stimuli, while triangle symbols indicate ‘tad’ stimuli. The sound name abbreviation is printed next to each data point, see Methods. Solid lines indicate the best linear fit. (**b**) The neural similarity between each novel sound and the template sounds is correlated with generalization performance on the gender ‘tad’ task. (**c**) The neural similarity between each novel sound and the template sounds is correlated with generalization performance on the voicing temporal compression task. (**d**) The neural similarity between each novel sound and the template sounds is correlated with generalization performance on the voicing multiple speaker task.
(PDF)

Figure S4 **Peak firing rate differences in high and low frequency neurons for gender and voicing distinctions.** (**a**) For the gender task using ‘dad’ stimuli, target female ‘dad’ sounds (red line) evoke a larger response in high frequency neurons compared to non-target male ‘dad’ sounds (blue line). Each of the 280 A1 recording sites from gender trained rats were binned by characteristic frequency into one of five bins each spanning one octave. Error bars indicate s.e.m. across each of the sounds. (**b**) For the gender task using ‘tad’ stimuli, target female ‘tad’ sounds evoke a larger response in high frequency neurons compared to non-target male ‘tad’ sounds. (**c**) For the voicing temporal compression task, target ‘dad’ sounds evoke a larger response in low frequency neurons compared to non-target ‘tad’ sounds. Each of the 168 A1 recording sites from voicing trained rats were binned by characteristic frequency into one of five bins each spanning one octave. (**d**) For the voicing multiple speaker task, target ‘dad’ sounds evoke a larger response in low frequency neurons compared to non-target ‘tad’ sounds.
(PDF)

Figure S5 **The percentage of sites responding at different onset latencies.** Each of the 441 A1 recording sites from experimentally naïve rats were binned by onset latency in response to tones. Sites were binned into one of five bins: sites responding faster than 10 ms, between 10 - 11 ms, 11- 12 ms, 12- 13 ms, or slower than 13 ms.
(PDF)

Figure S6 **Average percent of variance explained (R^2^) in anesthetized animals across the four generalization tasks using varying response windows.** (**a**) The average R^2^ across the 4 generalization tasks using a 30 -120 ms neural response analysis window is significantly correlated with generalization performance. Filled symbols indicate statistically significant correlations between neural similarity and behavior. (**b**) The average R^2^ across the 4 generalization tasks using a 50 ms analysis window with a varying start time. The correlation is strongest using the onset response information.
(PDF)

Figure S7 **Average percent of variance explained (R^2^) in awake animals across the four generalization tasks using varying response windows.** The average R^2^ across the 4 generalization tasks using a 50 - 60 ms neural response analysis window in awake animals is significantly correlated with generalization performance. Filled symbols indicate statistically significant correlations between neural similarity and behavior.
(PDF)

Text S1 (DOC)

## References

[1] Wyttenbach RA, May ML, Hoy RR (1996) Categorical perception of sound frequency by crickets. Science 273: 1542-1544. doi:10.1126/science.273.5281.1542. PubMed: 8703214. 8703214

[2] Seyfarth RM, Cheney DL, Marler P (1980) Monkey responses to three different alarm calls: evidence of predator classification and semantic communication. Science 210: 801-803. doi:10.1126/science.7433999. PubMed: 7433999. 7433999

[3] Shepard RN (1987) Toward a universal law of generalization for psychological science. Science 237: 1317-1323. doi:10.1126/science.3629243. PubMed: 3629243. 3629243

[4] Tversky A (1977) Features of Similarity. Psychol Rev 84: 327-352. doi:10.1037/0033-295X.84.4.327.

[5] Guttman N, Kalish HI (1956) Discriminability and stimulus generalization. J Exp Psychol Hum Learn 51: 79-88. PubMed: 13286444. 10.1037/h0046219 13286444

[6] Demany L (1985) Perceptual learning in frequency discrimination. J Acoust Soc Am 78: 1118-1120. doi:10.1121/1.393034. PubMed: 4031256. 4031256

[7] Pavlov IP (1927) Conditioned reflexes. Anrep G, translator; Anrep G, editor. London: Oxford University Press.

[8] Freedman DJ, Riesenhuber M, Poggio T, Miller EK (2001) Categorical representation of visual stimuli in the primate prefrontal cortex. Science 291: 312-316. doi:10.1126/science.291.5502.312. PubMed: 11209083. 11209083

[9] Kuhl PK, Miller JD (1975) Speech perception by the chinchilla: voiced-voiceless distinction in alveolar plosive consonants. Science 190: 69-72. doi:10.1126/science.1166301. PubMed: 1166301. 1166301

[10] Sandell JH, Gross CG, Bornstein MH (1979) Color categories in macaques. J Comp Physiol Psychol 93: 626-635. doi:10.1037/h0077594. PubMed: 113431. 113431

[11] Lisker L (1986) "Voicing" in English: a catalogue of acoustic features signaling /b/ versus /p/ in trochees. Lang Speech 29 (Pt 1): 3-11. 3657346 10.1177/002383098602900102

[12] Klatt DH, Klatt LC (1990) Analysis, synthesis, and perception of voice quality variations among female and male talkers. J Acoust Soc Am 87: 820-857. doi:10.1121/1.398894. PubMed: 2137837. 2137837

[13] Raphael LJ (2005) Acoustic cues to the perception of segmental phonemes. In: Pisoni DB Remez RE The Handbook of Speech Perception. Oxford: Blackwell Publishing.

[14] Francis AL, Kaganovich N, Driscoll-Huber C (2008) Cue-specific effects of categorization training on the relative weighting of acoustic cues to consonant voicing in English. J Acoust Soc Am 124: 1234-1251. doi:10.1121/1.2945161. PubMed: 18681610. 18681610 PMC2680590

[15] Schvartz KC, Chatterjee M (2012) Gender identification in younger and older adults: use of spectral and temporal cues in noise-vocoded speech. Ear Hear 33: 411-420. doi:10.1097/AUD.0b013e31823d78dc. PubMed: 22237163. 22237163 PMC3340495

[16] Ellis HD, Shepherd JW, Davies GM (1979) Identification of familiar and unfamiliar faces from internal and external features: some implications for theories of face recognition. Perception 8: 431-439. doi:10.1068/p080431. PubMed: 503774. 503774

[17] Wright J, Yang AY, Ganesh A, Sastry SS, Ma Y (2009) Robust face recognition via sparse representation. IEEE Trans Pattern Anal Mach Intell 31: 210-227. 19110489 10.1109/TPAMI.2008.79

[18] Brunelli R, Poggio T (1993) Face recognition: features versus templates. IEEE Trans Pattern Anal Machine Intell 15: 1042-1052. doi:10.1109/34.254061.

[19] Logan B, Salomon A (2001) A music similarity function based on signal analysis. Proceedings of IEEE International Conference on Multimedia and Expo. pp. 745-748.

[20] Haitsma J, Kalker T (2002) A highly robust audio fingerprinting system. Proceedings of the international Symposium on Music Information Retrieval.

[21] Bendor D, Wang X (2005) The neuronal representation of pitch in primate auditory cortex. Nature 436: 1161-1165. doi:10.1038/nature03867. PubMed: 16121182. 16121182 PMC1780171

[22] Steinschneider M, Fishman YI, Arezzo JC (2003) Representation of the voice onset time (VOT) speech parameter in population responses within primary auditory cortex of the awake monkey. J Acoust Soc Am 114: 307-321. doi:10.1121/1.1582449. PubMed: 12880043. 12880043

[23] Sharma A, Marsh CM, Dorman MF (2000) Relationship between N1 evoked potential morphology and the perception of voicing. J Acoust Soc Am 108: 3030-3035. doi:10.1121/1.1320474. PubMed: 11144595. 11144595

[24] Fishman YI, Reser DH, Arezzo JC, Steinschneider M (1998) Pitch vs. spectral encoding of harmonic complex tones in primary auditory cortex of the awake monkey. Brain Res 786: 18-30. doi:10.1016/S0006-8993(97)01423-6. PubMed: 9554938. 9554938

[25] Engineer CT, Perez CA, Chen YH, Carraway RS, Reed AC et al. (2008) Cortical activity patterns predict speech discrimination ability. Nat Neurosci 11: 603-608. doi:10.1038/nn.2109. PubMed: 18425123. 18425123 PMC2951886

[26] Perez CA, Engineer CT, Jakkamsetti V, Carraway RS, Perry MS et al. (2012) Different Timescales for the Neural Coding of Consonant and Vowel Sounds. Cereb Cortex, 23: 670–83. PubMed: 22426334. 22426334 10.1093/cercor/bhs045 PMC3563339

[27] Kawahara H (1997) Speech representation and transformation using adaptive interpolation of weighted spectrum: Vocoder revisited. Proceedings of the ICASSP 2 pp. 1303-1306.

[28] Perez CA, Engineer CT, Jakkamsetti V, Carraway RS, Perry MS et al. (2013) Different Timescales for the Neural Coding of Consonant and Vowel Sounds. Cereb Cortex 23: 670-683. doi:10.1093/cercor/bhs045. PubMed: 22426334. 22426334 PMC3563339

[29] Porter BA, Rosenthal TR, Ranasinghe KG, Kilgard MP (2011) Discrimination of brief speech sounds is impaired in rats with auditory cortex lesions. Behav Brain Res 219: 68-74. doi:10.1016/j.bbr.2010.12.015. PubMed: 21167211. 21167211 PMC3062672

[30] Ranasinghe KG, Vrana W, Matney C, Kilgard MP (2012) Neural Mechanisms Supporting Robust Discrimination of Spectrally and Temporally Degraded Speech. JARO. 10.1007/s10162-012-0328-1 PMC3387312 22549175

[31] Shetake JA, Wolf JT, Cheung RJ, Engineer CT, Ram SK et al. (2011) Cortical activity patterns predict robust speech discrimination ability in noise. Eur J Neurosci 34: 1823-1838. doi:10.1111/j.1460-9568.2011.07887.x. PubMed: 22098331. 22098331 PMC3286125

[32] Centanni TM, Engineer CT, Kilgard MP (2013) Cortical speech-evoked response patterns in multiple auditory fields are correlated with behavioral discrimination ability. J Neurophysiol 110: 177-189. doi:10.1152/jn.00092.2013. PubMed: 23596332. 23596332 PMC3727033

[33] Floody OR, Ouda L, Porter BA, Kilgard MP (2010) Effects of damage to auditory cortex on the discrimination of speech sounds by rats. Physiol Behav 101: 260-268. doi:10.1016/j.physbeh.2010.05.009. PubMed: 20580729. 20580729 PMC2910791

[34] Rennaker RL, Street S, Ruyle AM, Sloan AM (2005) A comparison of chronic multi-channel cortical implantation techniques: manual versus mechanical insertion. J Neurosci Methods 142: 169-176. doi:10.1016/j.jneumeth.2004.08.009. PubMed: 15698656. 15698656

[35] Miller GA, Nicely PE (1955) An Analysis of Perceptual Confusions Among Some English Consonants. J Acoust Soc Am 27: 338-352. doi:10.1121/1.1907526.

[36] Wright R (2004) A review of perceptual cues and cue robustness. In: Hayes B Kirchner R Steriade D Phonetically based phonology. Cambridge University Press.

[37] Peterson GE, Barney HL (1952) Control Methods Used in a Study of the Vowels. J Acoust Soc Am 24: 175-184. doi:10.1121/1.1906875.

[38] Shannon RV, Zeng FG, Kamath V, Wygonski J, Ekelid M (1995) Speech recognition with primarily temporal cues. Science 270: 303-304. doi:10.1126/science.270.5234.303. PubMed: 7569981. 7569981

[39] Xu L, Thompson CS, Pfingst BE (2005) elative contributions of spectral and temporal cues for phoneme recognition. 117: 3255-3267. 10.1121/1.1886405 PMC1414641 15957791

[40] Kluender KR, Diehl RL, Killeen PR (1987) Japanese quail can learn phonetic categories. Science 237: 1195-1197. doi:10.1126/science.3629235. PubMed: 3629235. 3629235

[41] Rosch E, Mervis CB (1975) Family resemblances: Studies in the internal structure of categories. Cogn Psychol 7: 573-605. doi:10.1016/0010-0285(75)90024-9.

[42] Hampton JA (1998) Similarity-based categorization and fuzziness of natural categories. Cognition 65: 137-165. doi:10.1016/S0010-0277(97)00042-5. PubMed: 9557381. 9557381

[43] Recanzone GH, Schreiner CE, Merzenich MM (1993) Plasticity in the frequency representation of primary auditory cortex following discrimination training in adult owl monkeys. J Neurosci 13: 87-103. PubMed: 8423485. 8423485 10.1523/JNEUROSCI.13-01-00087.1993 PMC6576321

[44] Gentner TQ, Margoliash D (2003) Neuronal populations and single cells representing learned auditory objects. Nature 424: 669-674. doi:10.1038/nature01731. PubMed: 12904792. 12904792 PMC2631575

[45] Weinberger NM, Bakin JS (1998) Learning-induced physiological memory in adult primary auditory cortex: receptive fields plasticity, model, and mechanisms. Audiol Neuro Otol 3: 145-167. doi:10.1159/000013787. PubMed: 9575382. 9575382

[46] Mesgarani N, David SV, Fritz JB, Shamma SA (2008) Phoneme representation and classification in primary auditory cortex. J Acoust Soc Am 123: 899-909. doi:10.1121/1.2816572. PubMed: 18247893. 18247893

[47] Thompson JV, Gentner TQ (2010) Song recognition learning and stimulus-specific weakening of neural responses in the avian auditory forebrain. J Neurophysiol 103: 1785-1797. doi:10.1152/jn.00885.2009. PubMed: 20107117. 20107117 PMC2853264

[48] Brown M, Irvine DR, Park VN (2004) Perceptual learning on an auditory frequency discrimination task by cats: association with changes in primary auditory cortex. Cereb Cortex 14: 952-965. doi:10.1093/cercor/bhh056. PubMed: 15115736. 15115736

[49] Reed A, Riley J, Carraway R, Carrasco A, Perez C et al. (2011) Cortical map plasticity improves learning but is not necessary for improved performance. Neuron 70: 121-131. doi:10.1016/j.neuron.2011.02.038. PubMed: 21482361. 21482361

[50] Schnupp JW, Hall TM, Kokelaar RF, Ahmed B (2006) Plasticity of temporal pattern codes for vocalization stimuli in primary auditory cortex. J Neurosci 26: 4785-4795. doi:10.1523/JNEUROSCI.4330-05.2006. PubMed: 16672651. 16672651 PMC6674162

[51] Wong SW, Schreiner CE (2003) Representation of CV-sounds in cat primary auditory cortex: intensity dependence. Speech Commun 41: 93-106. doi:10.1016/S0167-6393(02)00096-1.

[52] Engineer ND, Engineer CT, Reed AC, Pandya PK, Jakkamsetti V et al. (2012) Inverted-U function relating cortical plasticity and task difficulty. Neuroscience 205: 81-90. doi:10.1016/j.neuroscience.2011.12.056. PubMed: 22249158. 22249158 PMC3299820

[53] Takahashi H, Yokota R, Funamizu A, Kose H, Kanzaki R (2011) Learning-stage-dependent, field-specific, map plasticity in the rat auditory cortex during appetitive operant conditioning. Neuroscience 199: 243-258. doi:10.1016/j.neuroscience.2011.09.046. PubMed: 21985937. 21985937

[54] Buonomano DV, Merzenich MM (1995) Temporal information transformed into a spatial code by a neural network with realistic properties. Science 267: 1028-1030. doi:10.1126/science.7863330. PubMed: 7863330. 7863330

[55] Chang EF, Rieger JW, Johnson K, Berger MS, Barbaro NM et al. (2010) Categorical speech representation in human superior temporal gyrus. Nat Neurosci, 13: 1428–32. PubMed: 20890293. 20890293 10.1038/nn.2641 PMC2967728

[56] Russ BE, Orr LE, Cohen YE (2008) Prefrontal neurons predict choices during an auditory same-different task. Curr Biol 18: 1483-1488. doi:10.1016/j.cub.2008.08.054. PubMed: 18818080. 18818080 PMC2576490

[57] Tsunada J, Lee JH, Cohen YE (2011) Representation of speech categories in the primate auditory cortex. J Neurophysiol 105: 2634-2646. doi:10.1152/jn.00037.2011. PubMed: 21346209. 21346209 PMC3118748

[58] Blumstein SE, Stevens KN (1980) Perceptual invariance and onset spectra for stop consonants in different vowel environments. J Acoust Soc Am 67: 648-662. doi:10.1121/1.383890. PubMed: 7358906. 7358906

[59] Bertoncini J, Bijeljac-Babic R, Blumstein SE, Mehler J (1987) Discrimination in neonates of very short CVs. J Acoust Soc Am 82: 31-37. doi:10.1121/1.2024757. PubMed: 3624638. 3624638

[60] Formisano E, De Martino F, Bonte M, Goebel R (2008) "Who" is saying "what"? Brain-based decoding of human voice and speech. Science 322: 970-973. doi:10.1126/science.1164318. PubMed: 18988858. 18988858

[61] Staeren N, Renvall H, De Martino F, Goebel R, Formisano E (2009) Sound categories are represented as distributed patterns in the human auditory cortex. Curr Biol 19: 498-502. doi:10.1016/j.sbi.2009.05.005. PubMed: 19268594. 19268594

[62] Nelken I (2004) Processing of complex stimuli and natural scenes in the auditory cortex. Curr Opin Neurobiol 14: 474-480. doi:10.1016/j.conb.2004.06.005. PubMed: 15321068. 15321068

[63] Grace JA, Amin N, Singh NC, Theunissen FE (2003) Selectivity for conspecific song in the zebra finch auditory forebrain. J Neurophysiol 89: 472-487. PubMed: 12522195. 12522195 10.1152/jn.00088.2002

[64] Hernández A, Nácher V, Luna R, Zainos A, Lemus L et al. (2010) Decoding a perceptual decision process across cortex. Neuron 66: 300-314. doi:10.1016/j.neuron.2010.03.031. PubMed: 20435005. 20435005

[65] Hoffman HS (1958) Study of some cues in the perception of the voiced stop consonants. J Acoust Soc Am 30: 1035-1041. doi:10.1121/1.1909448.

[66] Eimas PD (1974) Auditory and linguistic processing of cues for place of articulation by infants. Atten, Percept, and psychphysics 16: 513-521. doi:10.3758/BF03198580.

[67] Dorman MF, Raphael LJ, Isenberg D (1980) Acoustic cues for a fricative-affricate contrast in word-final position. J Phon 8: 397-405.

[68] Reed P, Howell P, Sackin S, Pizzimenti L, Rosen S (2003) Speech perception in rats: use of duration and rise time cues in labeling of affricate/fricative sounds. J Exp Anal Behav 80: 205-215. doi:10.1901/jeab.2003.80-205. PubMed: 14674729. 14674729 PMC1284954

[69] Sinnott JM, Brown CH, Malik WT, Kressley RA (1997) A multidimensional scaling analysis of vowel discrimination in humans and monkeys. Percept Psychophys 59: 1214-1224. doi:10.3758/BF03214209. PubMed: 9401456. 9401456

[70] Walker KM, Bizley JK, King AJ, Schnupp JW (2011) Multiplexed and robust representations of sound features in auditory cortex. J Neurosci 31: 14565-14576. doi:10.1523/JNEUROSCI.2074-11.2011. PubMed: 21994373. 21994373 PMC3272412

[71] Walker KM, Schnupp JW, Hart-Schnupp SM, King AJ, Bizley JK (2009) Pitch discrimination by ferrets for simple and complex sounds. J Acoust Soc Am 126: 1321-1335. doi:10.1121/1.3179676. PubMed: 19739746. 19739746 PMC2784999

[72] Steinschneider M, Volkov IO, Fishman YI, Oya H, Arezzo JC et al. (2005) Intracortical responses in human and monkey primary auditory cortex support a temporal processing mechanism for encoding of the voice onset time phonetic parameter. Cereb Cortex 15: 170-186. PubMed: 15238437. 15238437 10.1093/cercor/bhh120

[73] Eggermont JJ (1995) Representation of a voice onset time continuum in primary auditory cortex of the cat. J Acoust Soc Am 98: 911-920. doi:10.1121/1.413517. PubMed: 7642830. 7642830

[74] Cano P, Batlle E, Kalker T, Haitsma J (2005) A Review of Audio Fingerprinting. J VLSI Signal Process 41: 271-284. doi:10.1007/s11265-005-4151-3.

[75] Freedman DJ, Riesenhuber M, Poggio T, Miller EK (2003) A comparison of primate prefrontal and inferior temporal cortices during visual categorization. J Neurosci 23: 5235-5246. PubMed: 12832548. 12832548 10.1523/JNEUROSCI.23-12-05235.2003 PMC6741148

[76] Romo R, Salinas E (2003) Flutter discrimination: neural codes, perception, memory and decision making. Nat Rev Neurosci 4: 203-218. doi:10.1038/nrn1058. PubMed: 12612633. 12612633

[77] Seger CA, Miller EK (2010) Category learning in the brain. Annu Rev Neurosci 33: 203-219. doi:10.1146/annurev.neuro.051508.135546. PubMed: 20572771. 20572771 PMC3709834

[78] Bidelman GM, Moreno S, Alain C (2013) Tracing the emergence of categorical speech perception in the human auditory system. NeuroImage 79: 201-212. doi:10.1016/j.neuroimage.2013.04.093. PubMed: 23648960. 23648960

[79] Ranasinghe KG, Vrana WA, Matney CJ, Kilgard MP (2013) Increasing diversity of neural responses to speech sounds across the central auditory pathway. Neuroscience, 252C: 80–97. PubMed: 23954862. 10.1016/j.neuroscience.2013.08.005 PMC3795858 23954862

[80] Chechik G, Anderson MJ, Bar-Yosef O, Young ED, Tishby N et al. (2006) Reduction of information redundancy in the ascending auditory pathway. Neuron 51: 359-368. doi:10.1016/j.neuron.2006.06.030. PubMed: 16880130. 16880130

[81] Russ BE, Ackelson AL, Baker AE, Cohen YE (2008) Coding of auditory-stimulus identity in the auditory non-spatial processing stream. J Neurophysiol 99: 87-95. PubMed: 18003874. 18003874 10.1152/jn.01069.2007 PMC4091985

[82] Selezneva E, Scheich H, Brosch M (2006) Dual time scales for categorical decision making in auditory cortex. Curr Biol 16: 2428-2433. doi:10.1016/j.cub.2006.10.027. PubMed: 17174917. 17174917

[83] Niwa M, Johnson JS, O'Connor KN, Sutter ML (2012) Activity related to perceptual judgment and action in primary auditory cortex. J Neurosci 32: 3193-3210. doi:10.1523/JNEUROSCI.0767-11.2012. PubMed: 22378891. 22378891 PMC3572866

[84] Niwa M, Johnson JS, O'Connor KN, Sutter ML (2013) Differences between primary auditory cortex and auditory belt related to encoding and choice for AM sounds. J Neurosci 33: 8378-8395. doi:10.1523/JNEUROSCI.2672-12.2013. PubMed: 23658177. 23658177 PMC3804137

[85] Bizley JK, Walker KM, Nodal FR, King AJ, Schnupp JW (2013) Auditory cortex represents both pitch judgments and the corresponding acoustic cues. Curr Biol 23: 620-625. doi:10.1016/j.cub.2013.03.003. PubMed: 23523247. 23523247 PMC3696731

[86] Cohen YE, Hauser MD, Russ BE (2006) Spontaneous processing of abstract categorical information in the ventrolateral prefrontal cortex. Biol Lett 2: 261-265. doi:10.1098/rsbl.2005.0436. PubMed: 17148378. 17148378 PMC1618918

[87] Gifford GW 3rd, MacLean KA, Hauser MD, Cohen YE (2005) The neurophysiology of functionally meaningful categories: macaque ventrolateral prefrontal cortex plays a critical role in spontaneous categorization of species-specific vocalizations. J Cogn Neurosci 17: 1471-1482. doi:10.1162/0898929054985464. PubMed: 16197700. 16197700

[88] Lomber SG, Malhotra S (2008) Double dissociation of 'what' and 'where' processing in auditory cortex. Nat Neurosci 11: 609-616. doi:10.1038/nn.2108. PubMed: 18408717. 18408717

[89] Pasley BN, David SV, Mesgarani N, Flinker A, Shamma SA et al. (2012) Reconstructing speech from human auditory cortex. PLOS Biol 10: e1001251 PubMed: 22303281. 22303281 10.1371/journal.pbio.1001251 PMC3269422

[90] Rauschecker JP, Scott SK (2009) Maps and streams in the auditory cortex: nonhuman primates illuminate human speech processing. Nat Neurosci 12: 718-724. doi:10.1038/nn.2331. PubMed: 19471271. 19471271 PMC2846110

[91] Rosenblatt F (1958) The perceptron: a probabilistic model for information storage and organization in the brain. Psychol Rev 65: 386-408. doi:10.1037/h0042519. PubMed: 13602029. 13602029

[92] Rumelhart DE, McClelland JL, University of California SDPRG (1986) Parallel Distributed Processing: Explorations in the Microstructure of Cognition. Cambridge, Massachusetts: MIT Press.

[93] Gütig R, Sompolinsky H (2006) The tempotron: a neuron that learns spike timing-based decisions. Nat Neurosci 9: 420-428. doi:10.1038/nn1643. PubMed: 16474393. 16474393

[94] Shepard RN (2004) How a cognitive psychologist came to seek universal laws. Psychon Bull Rev 11: 1-23. doi:10.3758/BF03206455. PubMed: 15116981. 15116981

[95] Deisseroth K (2011) Optogenetics. Nat Methods 8: 26-29. doi:10.1038/nmeth.f.324. PubMed: 21191368. 21191368 PMC6814250

[96] Boersma P (2001) Praat, a system for doing phonetics by computer. Glot International 5: 341-345.

[97] Sjölander K, Beskow J (2000) Wavesurfer - an open source speech tool. ICSLP 2000 Beijing, China.

