# Supplementary figures and images for: Similarity of Cortical Activity Patterns Predicts generalization Behavior
Source: PLoS One. 2013 Oct 16;8(10):e78607. doi: 10.1371/journal.pone.0078607 (PMC3797841; doi:10.1371/journal.pone.0078607)

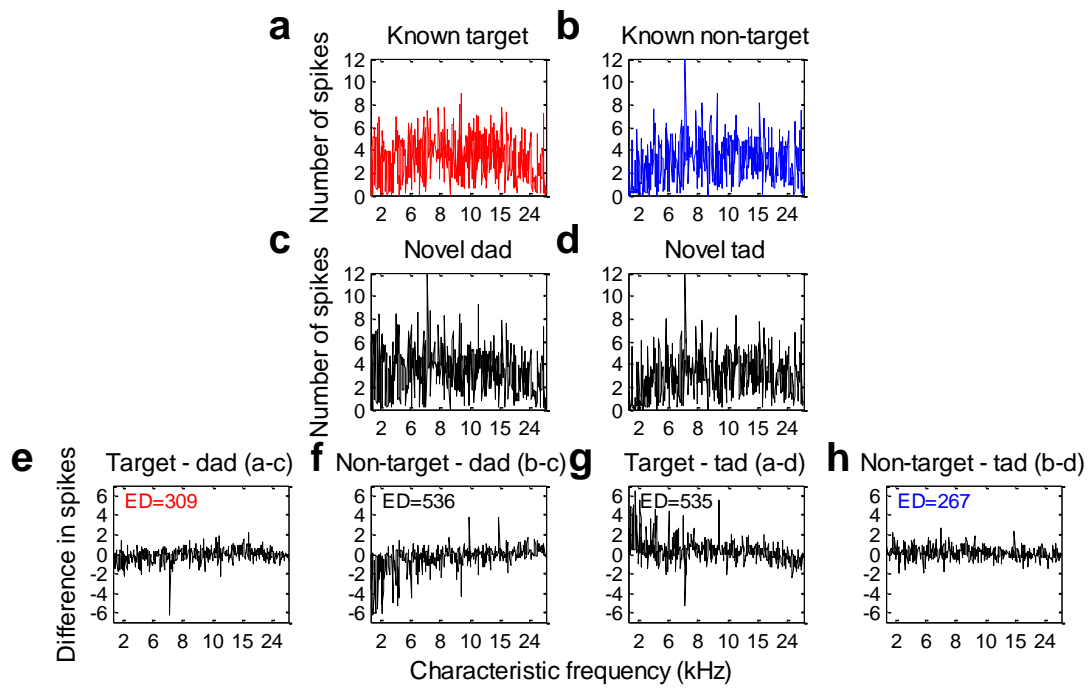

Supplement: Figure S1 — Neural similarity between two novel sounds and the trained target and trained non-target. Multi-unit data was collected from 441 recording sites (x axis) in eleven anesthetized rats and is ordered by the characteristic frequency (kHz) of each recording site. The number of spikes fired in response to each sound during the first 50 ms of the response is represented on the y axis. (a) The response to the known target sound (red, ‘dad’ spoken by female #1) and (b) known non-target sound (blue, ‘tad’ spoken by female #1). (c) The response to a novel ‘dad’ sound and a novel ‘tad’ sound (d). Both sounds were spoken by female #1 and temporally compressed by 50%. (e-h) The response pattern difference between the novel ‘dad’ sound and the target (e) and non-target sounds (f), and the novel ‘tad’ sound and the target (g) and non-target sounds (h). The difference between the novel ‘dad’ and the target (e, 309) was smaller than the difference between the novel ‘dad’ and the non-target (f, 536), indicating that the novel ‘dad’ and the target are more similar. The difference between the novel ‘tad’ and the non-target (h, 267) was smaller than the difference between the novel ‘tad’ and the target (g, 535), indicating that the novel ‘tad’ and the non-target are more similar. (PDF) [file pone.0078607.s001.pdf]

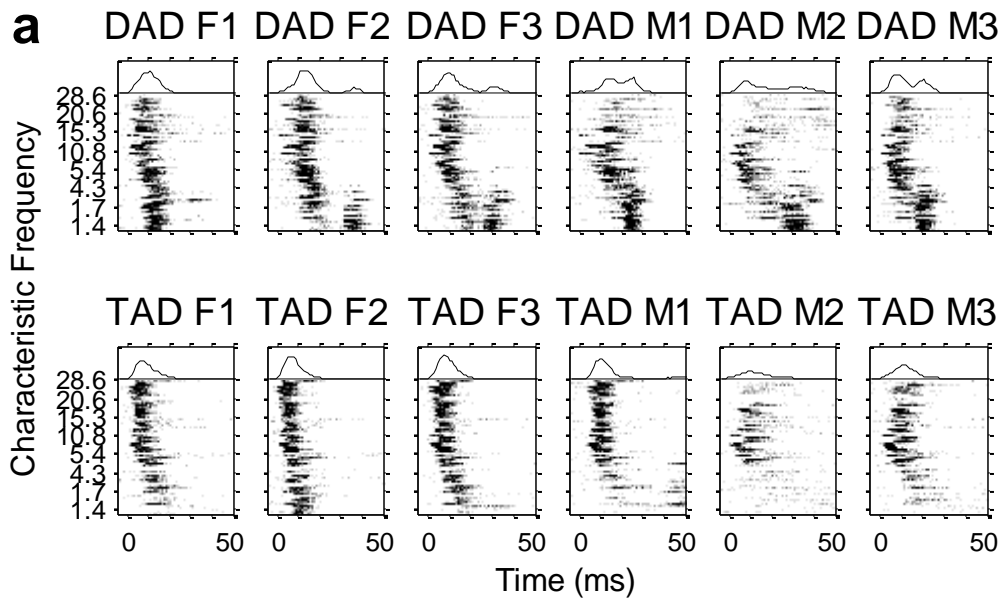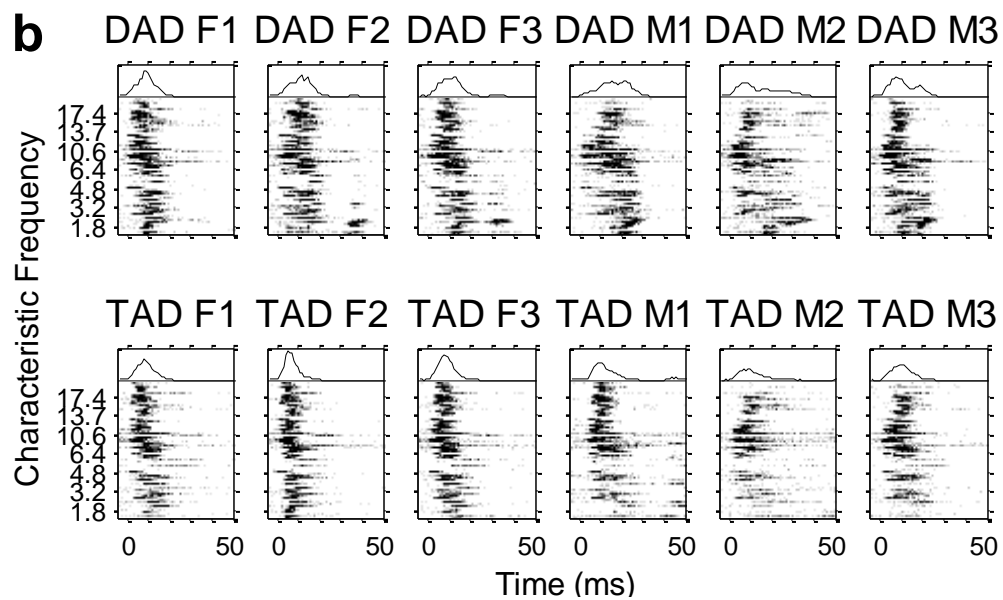

Supplement: Figure S2 — Neurograms depicting the onset response of gender trained and voicing trained rat A1 neurons. (a) Multi-unit data was collected from 280 recording sites in five anesthetized gender trained rats. Average post-stimulus time histograms (PSTH) derived from twenty repeats were ordered by the characteristic frequency (kHz) of each recording site (y axis). Time is represented on the x axis (-5 to 50 ms). The firing rate of each site is represented in grayscale, where black indicates 450 spikes/s. For comparison, the mean population PSTH evoked by each sound is plotted above the corresponding neurogram. To facilitate comparison between the naïve and trained responses, the mean PSTH y axis is set to 450 Hz for all neurogram figures. For gender trained rats, ‘tad’ female #3 evokes the maximum peak firing rate (330 Hz) across the twelve sounds. As in Figure 1, rows differ in voicing (top row is ‘dad’, bottom row is ‘tad’), while columns differ in gender (left three columns are female, right three columns are male). (b) Neurograms depicting the onset response of voicing trained rat A1 neurons to each of the twelve sounds shown in Figure 1. Multi-unit data was collected from 168 recording sites in four anesthetized voicing trained rats. For voicing trained rats, ‘tad’ female #2 evokes the maximum peak firing rate (414 Hz) across the twelve sounds. (PDF) [file pone.0078607.s002.pdf]

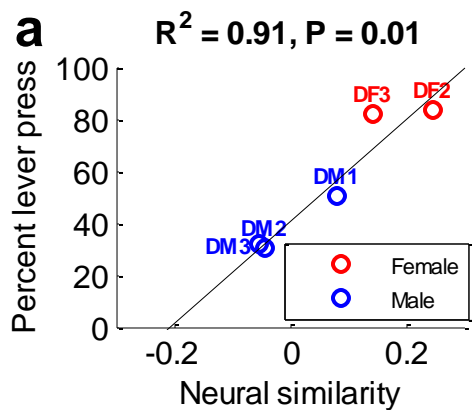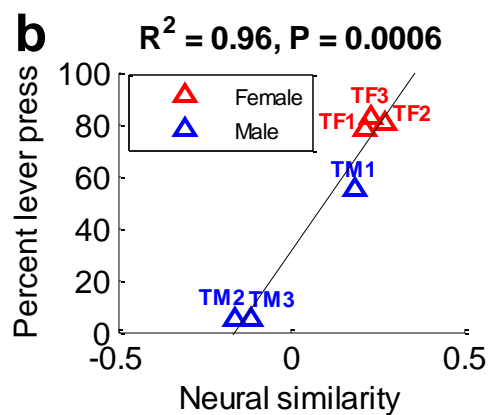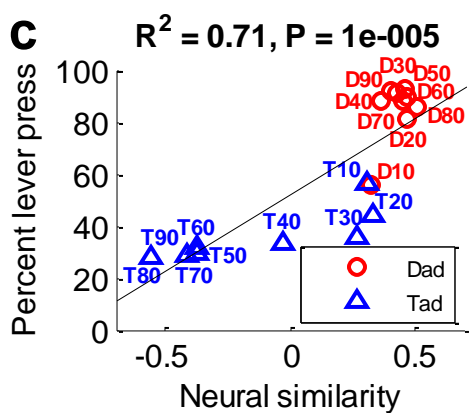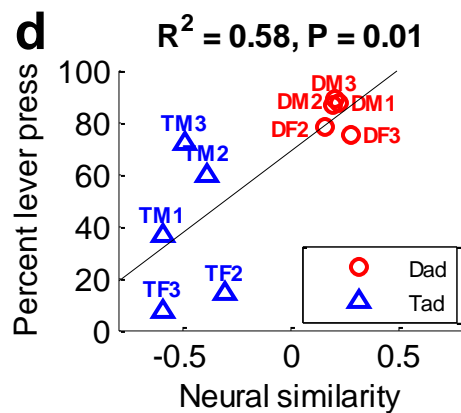

Supplement: Figure S3 — Neural correlates of generalization performance using neural responses from gender and voicing trained rats. (a) The normalized Euclidean distance (neural similarity) between the response pattern for each novel sound and the response pattern for each of the two template sounds is correlated with generalization performance on the gender ‘dad’ task. Positive values are more similar to the target template, while negative values are more similar to the non-target template. Red symbols represent target sounds and blue symbols represent non-target sounds. Circle symbols indicate ‘dad’ stimuli, while triangle symbols indicate ‘tad’ stimuli. The sound name abbreviation is printed next to each data point, see Methods. Solid lines indicate the best linear fit. (b) The neural similarity between each novel sound and the template sounds is correlated with generalization performance on the gender ‘tad’ task. (c) The neural similarity between each novel sound and the template sounds is correlated with generalization performance on the voicing temporal compression task. (d) The neural similarity between each novel sound and the template sounds is correlated with generalization performance on the voicing multiple speaker task. (PDF) [file pone.0078607.s003.pdf]

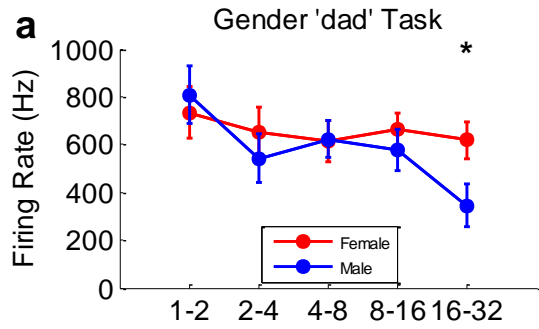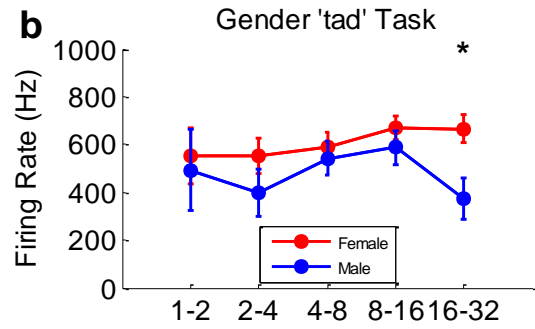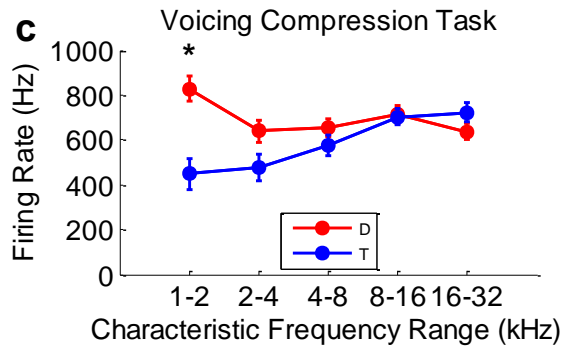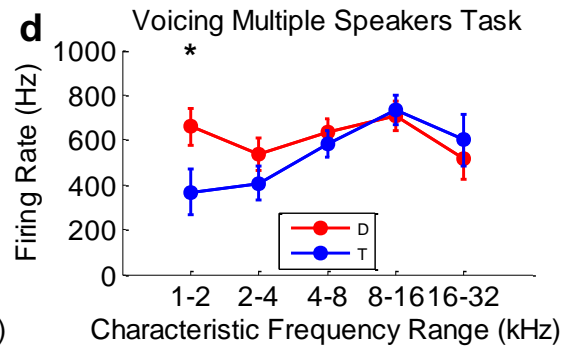

Supplement: Figure S4 — Peak firing rate differences in high and low frequency neurons for gender and voicing distinctions. (a) For the gender task using ‘dad’ stimuli, target female ‘dad’ sounds (red line) evoke a larger response in high frequency neurons compared to non-target male ‘dad’ sounds (blue line). Each of the 280 A1 recording sites from gender trained rats were binned by characteristic frequency into one of five bins each spanning one octave. Error bars indicate s.e.m. across each of the sounds. (b) For the gender task using ‘tad’ stimuli, target female ‘tad’ sounds evoke a larger response in high frequency neurons compared to non-target male ‘tad’ sounds. (c) For the voicing temporal compression task, target ‘dad’ sounds evoke a larger response in low frequency neurons compared to non-target ‘tad’ sounds. Each of the 168 A1 recording sites from voicing trained rats were binned by characteristic frequency into one of five bins each spanning one octave. (d) For the voicing multiple speaker task, target ‘dad’ sounds evoke a larger response in low frequency neurons compared to non-target ‘tad’ sounds. (PDF) [file pone.0078607.s004.pdf]

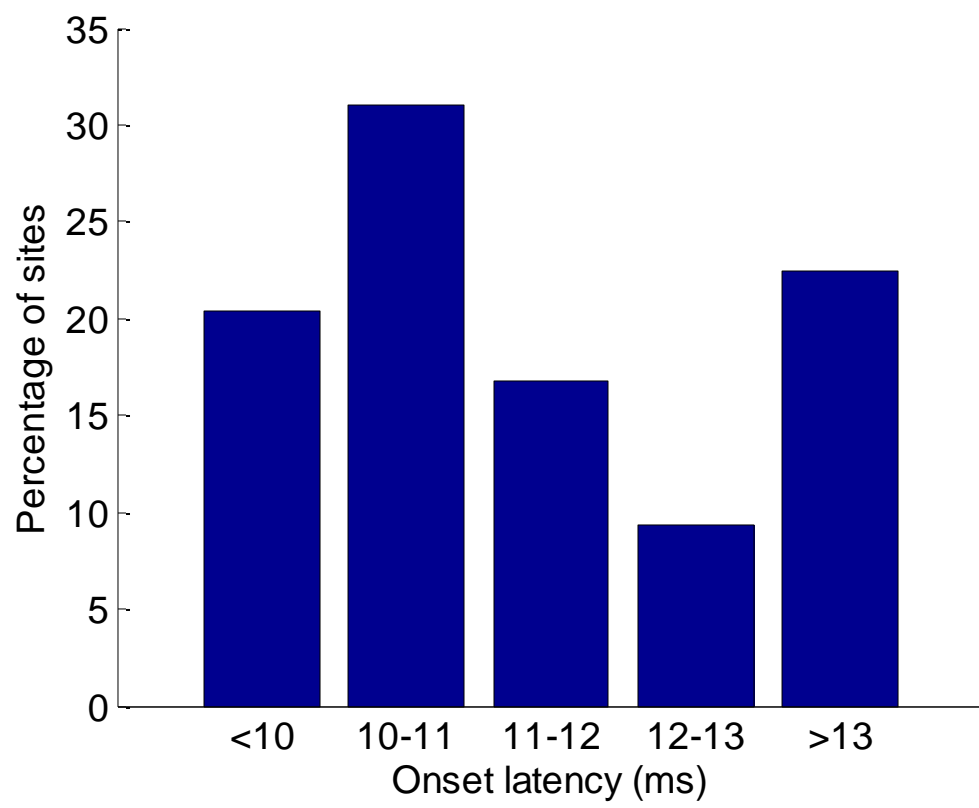

Supplement: Figure S5 — The percentage of sites responding at different onset latencies. Each of the 441 A1 recording sites from experimentally naïve rats were binned by onset latency in response to tones. Sites were binned into one of five bins: sites responding faster than 10 ms, between 10 - 11 ms, 11- 12 ms, 12- 13 ms, or slower than 13 ms. (PDF) [file pone.0078607.s005.pdf]

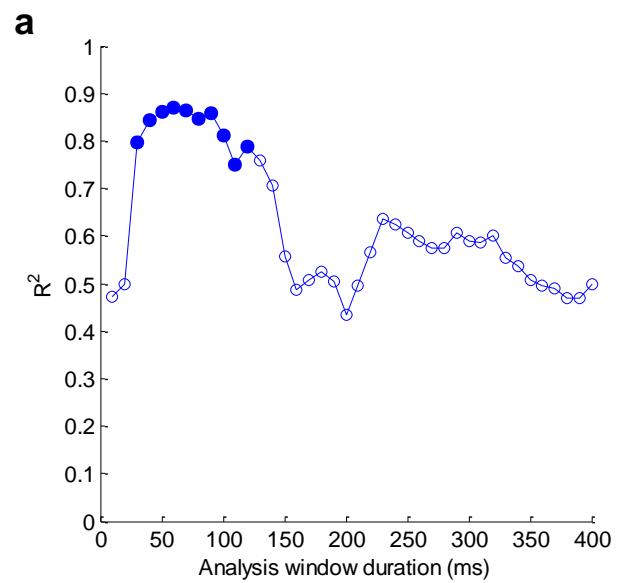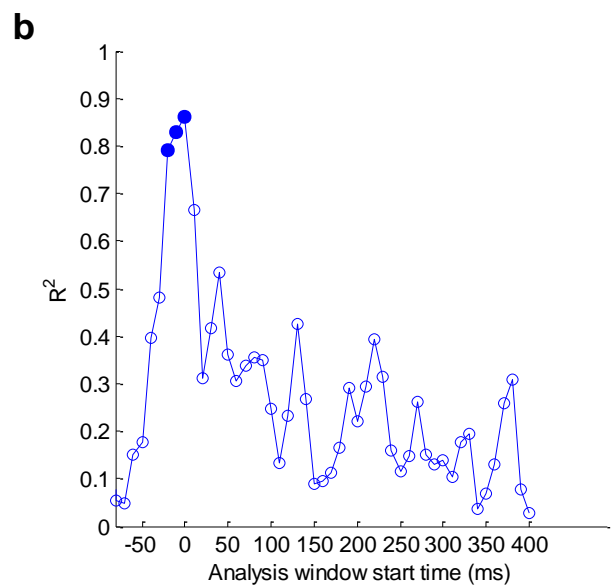

Supplement: Figure S6 — Average percent of variance explained (R2) in anesthetized animals across the four generalization tasks using varying response windows. (a) The average R2 across the 4 generalization tasks using a 30 -120 ms neural response analysis window is significantly correlated with generalization performance. Filled symbols indicate statistically significant correlations between neural similarity and behavior. (b) The average R2 across the 4 generalization tasks using a 50 ms analysis window with a varying start time. The correlation is strongest using the onset response information. (PDF) [file pone.0078607.s006.pdf]

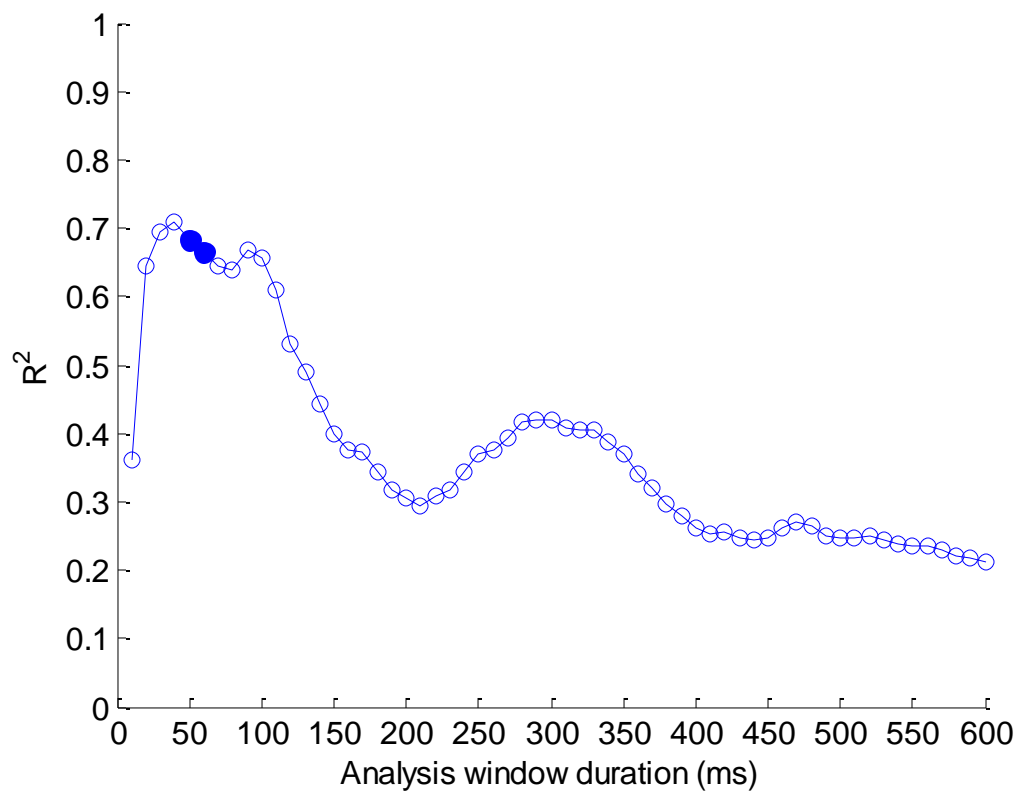

Supplement: Figure S7 — Average percent of variance explained (R2) in awake animals across the four generalization tasks using varying response windows. The average R2 across the 4 generalization tasks using a 50 - 60 ms neural response analysis window in awake animals is significantly correlated with generalization performance. Filled symbols indicate statistically significant correlations between neural similarity and behavior. (PDF) [file pone.0078607.s007.pdf]
