# Supplementary material for: Similarity of Cortical Activity Patterns Predicts generalization Behavior
Source: PLoS One. 2013 Oct 16;8(10):e78607. doi: 10.1371/journal.pone.0078607 (PMC3797841; doi:10.1371/journal.pone.0078607)
Supplement: Text S1 — (DOC) [file pone.0078607.s008.doc]

**Supporting information**

**Text S1**

Our hypothesis that generalization is based on similarity to the trained templates suggests that in order to accurately generalize to novel stimuli the trained sounds must provide a clear indication of what separates members of the different categories. We predicted that if the training sounds differ in more than one salient cue (i.e. so that the sounds could be categorized in more than one way), then generalization would be impaired because subjects would not know which cues reflect category differences and which do not. To test this prediction, we trained ten rats to discriminate between a female ‘dad’ and a male ‘tad’ (Figure 1) and then tested them on tasks requiring them to categorize speech sounds by gender (n = 5 rats) or voicing (n = 5 rats). The voicing group was rewarded for lever pressing to ‘dad’ sounds and the gender group was rewarded for lever pressing to female speakers. Training occurred until each rat reached a minimum criteria of a d’ ≥ 1.5 for 2 sessions (average of 3.2 ± 0.7 days), after which the rats were tested for their ability to correctly categorize a set of ten novel ‘dad’ and ‘tad’ sounds spoken by six speakers. When the training stimuli differed in two features (gender and voicing, a female ‘dad’ vs. a male ‘tad’), the number of sessions required to reach performance criteria was significantly shorter compared to the previous Voicing Task and Gender Task groups of rats (3.2 days vs. 9.3 days, p < 0.0001). Performance on the day before generalization testing was not significantly different from the previous Voicing Task and Gender Task groups. (d’ = 1.7 vs. d’ = 1.8, p = 0.51). As expected, the rats trained on ill-defined categories were only slightly above chance performance on the first day of generalization testing (d’ = 0.4, p = 0.034 for rats required to categorize by gender; d’ = 0.5, p = 0.06 for rats required to categorize by voicing). This level of performance was significantly worse than the first day performance of rats in the Voicing Task or Gender Task groups (d’ = 1.5, p < 0.0001). This finding supports our hypothesis that exemplars that provide a clear indication of what separates categories results in the most accurate generalization and is consistent with psychophysical studies (Allen and Brooks, 1991; Iverson et al., 2003).

After two weeks of testing, both groups of rats trained on ill-defined categories learned to accurately categorize the sounds (voicing d’ = 0.99, p = 0.02; gender d’ = 1.5, p = 0.006). As predicted from the previous Voicing Task and Gender Task groups, neural similarity was highly correlated with last day categorization performance on both tasks (voicing R^2^ = 0.71, p = 0.002; gender R^2^ = 0.95, p < 0.0001), and using trained neural responses did not significantly improve the correlation (voicing R^2^ = 0.74, p = 0.001; gender R^2^ = 0.91, p < 0.0001). The Euclidean distance between the test stimuli was not increased in either group compared to one another or naïve rats (p = 0.68). Collectively, these results support our hypothesis that the similarity of activity patterns in sensory cortex supports effective stimulus categorization and that plasticity in A1 is not necessary for improved stimulus categorization.
